# Antiphospholipid antibodies: Paradigm in transition

**DOI:** 10.1186/1742-2094-6-3

**Published:** 2009-01-20

**Authors:** Lawrence L Horstman, Wenche Jy, Carlos J Bidot, Yeon S Ahn, Roger E Kelley, Robert Zivadinov, Amir H Maghzi, Masoud Etemadifar, Seyed Ali Mousavi, Alireza Minagar

**Affiliations:** 1 Wallace Coulter Platelet Laboratory, Division of Hematology and Oncology, Department of Medicine, Miller School of Medicine, University of Miami, Miami, Florida, USA; 2 Department of Neurology, Louisiana State University Health Sciences Center, Shreveport, LA 71130, USA; 3 Buffalo Neuroimaging Analysis Center, The Jacobs Neurological Institute, Department of Neurology, School of Medicine and Biomedical Sciences, State University of New York at Buffalo, Buffalo NY, USA; 4 Department of Neurology, Isfahan University of Medical Sciences, Isfahan, Iran

## Abstract

**Objectives:**

This is a critical review of anti-phospholipid antibodies (aPL). Most prior reviews focus on the aPL syndrome (APS), a thrombotic condition often marked by neurological disturbance. We bring to attention recent evidence that aPL may be equally relevant to non-thrombotic autoimmune conditions, notably, multiple sclerosis and ITP.

**Organization:**

After a brief history, the recent proliferation of aPL target antigens is reviewed. The implication is that many more exist. Theories of aPL in thrombosis are then reviewed, concluding that all have merit but that aPL may have more diverse pathological consequences than now recognized. Next, conflicting results are explained by methodological differences. The lupus anticoagulant (LA) is then discussed. LA is the best predictor of thrombosis, but why this is true is not settled. Finally, aPL in non-thrombotic disorders is reviewed.

**Conclusion:**

The current paradigm of aPL holds that they are important in thrombosis, but they may have much wider clinical significance, possibly of special interest in neurology.

## Background

This manuscript critically compares the many theories and concepts of anti-phospholipid antibodies (aPL) as they pertain to the antiphospholipid syndrome (APS) and other clinical conditions where they occur. This review is not primarily concerned with clinical diagnosis and management, except peripherally. Although the topic of aPL has been reviewed many times, this review was inspired by findings in our laboratory and others suggesting that aPL may play roles in a variety of disorders apart from APS, not necessarily thrombotic.

According to Eng [1] and others, it was Pangborn who in 1941, following Wasserman's test for syphilis in 1903, identified an acidic phospholipid (PL) as the apparent target antigen of the test, specifically, cardiolipin (CL). CL is named for the bovine heart muscle from which it was obtained, heart being rich in mitochondria, a main source of CL. In 1952, Conley and Hartmann first described the lupus anticoagulant (LA), later interpreted as a consequence of aPL, in association with a hemorrhagic diathesis [2]. However, this and other early clinical observations were later overshadowed by frequent findings of thrombosis associated with positive anti-CL (aCL) test, leading to recognition of the aPL syndrome (APS) in the 1980s by Harris et al [3,4] and by Hughes et al [5], originally called anticardiolipin (aCL) syndrome, now sometimes Hughes' syndrome.

Although diagnostic criteria vary somewhat depending on sources, APS is generally defined by a repeatedly positive test for one or more aPL in conjunction with thrombosis or recurring pregnancy loss [6-13]. It is often accompanied by thrombocytopenia, episodic neurological disturbances [14], and/or numerous other clinical manifestations [15]. APS may be secondary to other underlying conditions, notably systemic lupus erythematosus (SLE); otherwise, in the absence of other disorders is known as primary APS (PAPS). In its most life-threatening form, it is known as catastrophic APS (CAPS). In patients with CAPS occlusion of small blood vessels leads to multi-organ failure. Many reviews of APS with focus on clinical manifestations and management, laboratory methodologies, and hypotheses to account for the association between aPL and thrombosis exist [16-22]. However, as stressed in this review, many uncertainties remain.

### What are aPL and how are they measured?

Originally, aPL were defined as antibodies reacting to cardiolipin (CL) but for reasons discussed below, no widely accepted definition of aPL any longer exists. They are measured by two distinct kinds of tests, solid-phase for particular aPL, and coagulation-based for LA. The former is usually an enzyme-linked immunosorption assay (ELISA), consisting in outline of adding a sample of patient serum or plasma to a plastic well coated with some particular PL or mixture of PLs, with or without a specific protein cofactor (see below), then measuring how much patient immunoglobulin (Ig) is captured by adding an anti-human IgG, IgM, or IgA conjugated with an enzyme that generates a colored product. Despite its simplicity, this procedure is subject to many subtle variations which can grossly affect results, discussed later. In contrast, LA are detected by the prolonged time required for coagulation of the patient's plasma relative to normal plasma in a test designed to be sensitive to PL. Most commonly, the dilute Russell viper venom time (dRVVT) is used. It is widely believed that the prolongation is caused by an aPL occupying sites on the PL which are required for binding the coagulation factors, thereby prolonging the time. The LA is discussed later.

### Protein cofactors and definition of aPL

According to Roubey [16], three groups independently in 1990 demonstrated that a positive ELISA test for aCL depended on a protein cofactor, β_2_-glycoprotein-I (β_2_GPI) [23-25]. This had gone unnoticed because β_2_GPI is present in most ELISA methods, either in the bovine or other animal serum commonly used for dilutions and/or for blocking the plate against non-specific binding, or in the test serum or plasma. The requirement for β_2_GPI can be shown by using purified Ig and excluding other sources of β_2_GPI. As a result, it was often argued that anti-β_2_GPI is equivalent to aCL [26-29] or is even a surrogate for aPL generally [30]. Among the diagnostic criterion of APS has been the presence of β_2_GPI-dependent aCL [9]. Since then, many additional aPL cofactors have been identified, reviewed below. This has resulted in the widely held opinion that all clinically relevant aPL are directed against protein target antigens rather than any particular pure PL. If so, the term aPL is an outmoded misnomer [31]. Thus, some authors place aPL in quotation marks to indicate the fallacy [32]. Accordingly, the implicit working definition of aPL now appears to be *an antibody that targets a PL-binding protein *[31-33]. The aPL may react preferentially with the PL-bound form, or may bind to the free antigen in plasma as an immune complex (IC) to potentiate binding to a given PL.

### Do all clinically relevant aPL target proteins?

On the other hand, it is known that antibodies reacting to pure PL (no protein cofactor requirement) do occur in many infectious diseases, classically in syphilis but also in leprosy, leishmaniasis, malaria, Epstein Barr virus, hepatitis C virus and HIV, e.g. [34,35]. These are spoken of as the infectious disease type of aPL, and are often said to be non-pathogenic in themselves and thus clinically irrelevant. Partly for this reason, at least one updated criterion for APS diagnosis has dropped the requirement for aCL testing entirely [36]. However, Nash et al found that omitting classical aCL assay caused 25% of APS patients to be missed [37], and therefore urged that aCL testing be retained. Two commentaries on that article concur [38,39] and cite additional reasons.

More generally, the view that all clinically relevant aPL are directed against proteins has been challenged by a number of authorities [40-43]. To the examples cited by those authors we may cite the study of von Landenberg et al [44] in which an IgG antibody was cloned from B cells of each of two patients, one from APS with thrombosis and one from SLE without thrombosis, both of which reacted with CL yet showed no requirement for any protein in normal serum. Sorice et al [45] gave evidence that aCL and anti-β_2_GPI are distinctly different antibodies, as did Forastiero et al. [27]; but the operative question debated is whether they are "clinically relevant". Findings concerning aCL in HIV further emphasize uncertainties about protein cofactor-independent aPL [46] (discussed later). McIntryre et al. provide criteria for distinguishing protein-dependent and -independent aPL [42]. More recently, biosensor analysis looks promising for clarification [47].

There is another and quite different justification for continued use of the term, aPL. Many laboratories, including ours, routinely test patients against a panel of pure PL. If positive, it is most likely that an antibody against some PL-binding protein in serum is being detected. However, since the identity of that putative protein is unknown, there is little choice but to speak of it as aPL+.

### Survey of the antigens (or "cofactors")

Table 1 lists most of the aPL target antigens commonly recognized and several that are less well recognized as such, although meeting the above definition [48-68]. They are listed in groups from the most well-recognized and studied at top to less familiar ones at bottom.

**Table 1:** Antigens of antiphospholipid antibodies

**Antigen**	**References**	
Group 1. Best established & studied		

β2GPI	Many, e.g.	29, 48,49

Prothrombin	Many, e.g.	8, 98, 50

Protein C, S	Many, e.g.	93

Annexin V	Many, e.g.	11, 123,124

Group 2. Also accepted but less studied		

Thrombin		141

Annexin 2		130, 140

Complement C4, FH		131–133

Kininogens		31, 51,52,143

Kallikrein-related		52,133

FVII/FVIIa		135

Antithrombin III		137,138

Group 3. Pure phospholipids		

Cardiolipin (CL)	Many, e.g.	46

PE	Many, e.g.	31

PS, PC, etc.	Many, e.g.	53

Oxidized CL		54,55

Oxidized LDL, other PL.		56–58,147,148

Group 4. Often or sometimes included		

Plasmin		128

Tissue factor (TF)		134

TF path. inhibitor (TFPI)		130, 309

TPA		60,129

Platelet activating factor (PAF)		153

CD40/CD40L		154

Group 5. Associated or candidate aPL		

CD36		157,158

Thrombomodulin		32

EPCR		61,63

Phospholipase A2		156

Group 6. Implicit under definition		

FVIII	Many, e.g.	63–66,141

FX		16

FXI		52,67,155

FXII		68,161

#### Anti-β_2_-glycoprotein I (anti-β_2_GPI)

As already mentioned, anti-β_2_GPI has been equated with aCL, so that β_2_GPI (a.k.a. apolipoprotein H) became the most widely accepted and studied antigen relevant to APS [26,29,32]. However, several later studies found only a weak association between anti-β_2_GPI and thrombosis [69,70], and this is the main reason why the aCL or β_2_GPI-dependent aCL test has been dropped as a criterion of APS [36,70]. On the other hand, several studies have shown a strong association provided anti-β_2_GPI is at very high titer [71-73]. The role of anti-β_2_GPI in the LA test is reviewed later.

Despite the weak association, β_2_GPI has been the main focus of theories to account for APS [22], discussed later. Evidence suggests that pathogenic anti-β_2_GPI are limited to specific epitopes, especially the amino terminal (domain 1) [74-76]. The natural function of β_2_GPI is unclear but it may contribute to regulating fibrinolysis [77] and platelet function [78]. β_2_GPI co-purifies with thrombospondin from platelets [79]. It exhibits modest anticoagulant effects [80-83] which are augmented when bound to antibody [11]. However, Pengo has indicated that anti-β_2_GPI can have either anti- or pro-coagulant effects [84]. Arvieux found that oxidation of β_2_GPI caused either enhanced or decreased binding to Ig: 10 of 20 patients enhanced, 10 decreased [85].

One leading theory to account for its thrombogenicity is that by binding to PL surfaces, it interferes with the anticoagulant protein C system [86]. This popular theory has several variants. Roubey listed six variant theories for the procoagulance of β_2_GPI as of 1994 [32], all seeming viable. β_2_GPI and anti-β_2_GPI have been localized to late endosomes in cytoplasm as well as the endothelial cell surface [87]. Since the literature on β_2_GPI is large and is well summarized in reviews cited above, it is not further discussed here.

#### Anti-protein C, S (aProtC, aProtS)

These proteins, together with thrombomodulin, protein C inhibitor, and the endothelial protein C receptor (EPCR), constitute a vital natural anticoagulant system [88,89]. Deficiency of protein S, for example, is both a risk factor and an explanation for thrombotic events, either familial [90] or by acquired Ab [91], or by other causes [92]. The FV Leiden mutation is a risk factor by making FVa resistant to inactivation by activated protein C. Accordingly, aPL against ProtC and/or ProtS have been proposed to account for or contribute to thrombosis in APS [93]. It has been shown that β_2_GPI enhances the function of ProtS [94], suggesting that anti-β_2_GPI impairs the function of ProtS. However, these Ab are less common than anti-β_2_GPI in most studies of APS. Bick and Pegram [95] provide a survey of the many kinds and causes of defects of ProtC and ProtS, including aPL. Duchemin et al detected LA in 85% of a group of 17 children with varicella, and aProtS was present in 75% of them, and thrombosis in 24%, all LA+ [96]. In a large study by Nojima et al, neither aProtS nor aProtC was associated with arterial thrombosis but aProtS exclusively associated with venous thrombosis (p = 0.003) while anti-β_2_GPI had no such association [97].

#### Anti-prothrombin (aPT)

The majority of reports and reviews now discount the clinical value of assay for aPT [69,98,99]. At the same time, most concur that its wide prevalence and intriguing relationships warrant further study. Pengo et al found strong association of thrombosis with anti-β_2_GPI but not with aPT and conclude that aPT is not a marker of thrombosis [100]. Indeed, it has been reported that aPT often has a hemorrhagic diathesis [101]. Conversely, Pasquier et al found significant association between venous thromboembolism (VTE) and aPT but not with anti-β_2_GPI or other assays in their study. However, after patients were stratified by additional risk factors; they concluded that aPT screening has little value [102]. Of interest, however, is that aPT strongly correlated with aProtC and aProtS [100]. On the other hand, Vaarala et al found good association of aPT with myocardial infarcts in middle-aged men [103]. A review of aPT [8] suggests that interest in aPT stemmed, in part, from its occurrence in 75% of LA-positive patients [104] (see later on LA). Galli et al found that aPT are heterogeneous [105].

Atsumi et al [106] confirmed that aPT are heterogeneous (as are most aPL) and that the clinical relevance of aPT depends on the method of detection. For example, the complex of aPT with phosphatidylserine (aPT/PS) was associated with thrombosis but aPT itself was not. This highlights the importance of methodologies, discussed later. They, too, confirmed that aPT/PS correlates closely with LA. Donohoe et al explored variations of aPT methods and found conditions where aPT IgM, but not IgG, significantly associated with thrombosis [107]. Thus, aPT may indeed have real clinical significance but only if assayed in certain ways, and includes IgM assay. The review cited above [8] concludes cautiously, and notes that aPT occurs in several inflammatory disorders besides APS [108]; but this is true of many aPL.

#### Anti-annexin V (aAnV)

AnV, a calcium-dependent PL membrane-binding protein, was first identified in abundance in placenta, resulting in it being called placental anticoagulant protein 1. Other synonyms include lipocortin, anchorin, calphobindin and vascular anticoagulant alpha [109]. Otherwise, its function is not clear, but it does act like an ion channel [110]. It is widely distributed but at lower concentrations than in pregnancy. AnV binds specifically to anionic PL, especially PS, and therefore acts *in vitro *as an anticoagulant by competing for sites on PL membranes where the coagulation factors normally assemble into active complexes (prothrombinase and tenase) and so mimics the LA effect [111,112]. Fluorescent AnV is widely used to identify PS-positive apoptotic cells, procoagulant microparticles, and activated cells [113-117]. It has been shown that PS triggers a specific receptor for phagocytosis [118], although, as noted in that commentary, details are complex.

Antibodies to AnV (aAnV) are widely accepted as aPL in most reviews, e.g. [119]. In one study, frequency of aAnV+ in SLE, APS and other prothrombotic disorders was the same as anti-β_2_GPI about 30%, and was the only significant risk factor for recurrent fetal loss, p = 0.01 [97]. Since AnV is believed to be an important anticoagulant during pregnancy, Donohoe et al offer evidence that anti-β_2_GPI, along with blockade of AnV by aAnV, may explain APS-associated miscarriage [120]. Rand et al had earlier proposed that aPL caused pregnancy loss by reducing levels of AnV [121]. The association of aAnV with miscarriage has been observed frequently, recently by Galli et al [122]. Anti-AnV has been studied extensively in SLE [123-125] including in comparison with other aPL [97]. Like most aPL, aAnV has been noted in other conditions, notably in rheumatoid arthritis, where it may play a role in pathology [126,127]. In that connection, it may be added that variants of AnV occur, even within a single tissue such as cartilage [128], which may help explain discordant findings. Binding of AnV is sensitive to the structure of the PL surface [129]. Zhang et al reported that endothelial activation induced by aPL is mediated by AnV [130]. Several workers have proposed a role of aAnV in LA activity, discussed later.

#### Group 2 of Table 1

No attempt is made to review all the aPL listed. Group 2 includes those commonly cited in review articles but which are less well studied than Group 1. Anti-kininogens are discussed below with anti-phosphatidyl ethanolamine (aPE). Kertesz et al found anti-complement factor H (aCFH) competitive with anti-β_2_GPI for CL binding [131]. Similarly, Arnout detected aCFH along with anti-β_2_GPI in all of 5 plasma samples [132], and this was confirmed again by Rampazzo et al, who also found anti-C4 (aC4) [133]. Hemolytic uremic syndrome (HUS) of the non-*E. coli *type is thought to be mediated by aCFH, e.g. [134].

Anti-factor VII/VIIa (aFVII) was regularly found in aPL ELISA assays by Bidot et al [135]. Later, Minagar et al found a close association of aFVII with specific clinical states in multiple sclerosis [136], as discussed later.

Antibodies to antithrombin III (aAT-III) in APS patients were reported by Kolev et al [137], and were found to be associated with anti-heparin Ab in APS [138], i.e., heparin-induced thrombocytopenia (HIT). Arnout has drawn interesting parallels between aPL and HIT/anti-platelet factor 4 [139]. The recent discovery of anti-annexin 2 in 40% of APS patients [140] aroused much interest, as did the finding of anti-thrombin Ab [141,142].

#### Group 3 of Table 1: Pure phospholipids (PL)

Aside from CL, the most commonly tested PL are phosphatidylcholine (PC), phosphatidylserine (PS), and phosphatidyl ethanolamine (PE), sometimes in mixtures; e.g. [119,135]. It appears that the protein cofactors responsible for positive aPE are predominately kininogens or kininogen-IC [143]. McIntyre et al proposed a novel hypothesis of aPL-dependent thrombosis on that basis [31]. Sanmarco et al reported a strong association of aPE IgM, but not other aPL, with APS and other unexplained thromboses [144]; however, an earlier report found that aPE was the only aPL tested that was *not *associated with thrombosis [145]. This further illustrates frequent discrepant findings. In a study of LA+ patients, who were divided into drug-induced *vs. *auto-Ab associated, aPE was 95% positive in the autoimmune groups (SLE, APS) [146].

As referenced in Table 1, much interest has centered on Ab to oxidizied PL (aOxPL) or oxidized lipoproteins such as low-density lipoproteins (LDL) in relation to thrombosis. This topic has extensive literature and we will only give some examples. Wu et al [147] followed 2322 subjects, age 50 or older, for up to 20 years. The investigators found that anti-oxLDL IgA correlated closely with aCL (p < 0.0001) but IgM and IgG had weak or no association, respectively. Steinerova et al [148] measured anti-oxPL in infants and observed that breast-fed babies, during the first 3 months, had much lower levels (p < 0.001) and less DNA breaks than those not breast-fed. As noted later, CL easily oxidizes in air so that many reports actually measure aOxCL, not aCL.

#### Group 4 of Table 1

These are PL membrane-binding proteins against which auto-Ab are known to occur, and therefore meet the definition of aPL, yet are not commonly listed in reviews of aPL, even though their binding properties suggest they may be detected, if unwittingly, in aPL assays against pure PL. However, a recent aPL review [22] includes Ab to plasmin [149] and to tissue plasminogen activator (aTPA) [150]. Tissue factor pathway inhibitor (TFPI) associates with many PL, and high-titer aTFPI was reported in APS [151]. (It was reported that anti-β_2_GPI suppresses TFPI activity [152].) Certainly aTFPI is a candidate for prothrombotic effects but is rarely tested for.

Anti-platelet activating factor (aPAF) has also been reported in APS [153], as has anti-CD40 [154]. Tissue factor itself (TF), which is known to be carried on PS-expressing cell-derived microparticles, is also capable of eliciting auto-Ab, and aTF was listed as an aPL in at least one review [33], citing De Groot [155]. According to De Groot's review, anti-phospholipase A2 was also regarded as an aPL, by Vermylen and Arnout, 1992; and see [156].

#### Group 5 of Table 1

These are agents which do not fit the definition since they are normally membrane-bound, not free in plasma, yet have been listed in relation to aPL, if not aPL per se. Roubey [32] included anti-thrombomodulin (aTM) and anti-heparan sulfate proteoglycans (aHSP) "because of their respective roles in the control of thrombosis". Likewise, antibodies to CD36 (a.k.a. platelet glycoprotein IV) have been implicated in APS [157,158] and cited in aPL review [16], as has aEPCR, which can exist in soluble form. Anti-ADAMTS13 [159], believed responsible for most cases of TTP, is also prothrombotic; but these clearly fall outside the definition of aPL.

#### Group 6 of Table 1

These are PL-binding coagulation factors known to sometimes elicit autoantibodies but are not usually recognized as aPL. This is inconsistent with recognition of anti-prothrombin (aPT), anti-thrombin, anti-protein C, anti-FVII, anti-kininogens and anti-tPA as bona fide aPL. They can be detected in some aPL assays against pure PL if plasma is used and Ca^2+^present. Some have been implicated in APS. For example, Gallimore et al, after studies to measure FXII deficiency in the confounding presence of aPL [160], later recognized that anomalous effects were actually due to anti-FXII, leading to the discovery that anti-FXII is specifically associated with recurrent fetal loss [161].

Deficiency of FVIII is the hallmark of hemophilia A but efforts to correct it with FVIII concentrates are plagued by the rise of anti-FVIII in response. Of interest, the anti-FVIII is often associated with aPL/LA, as in the report of Nuss et al, confirming prior reports in finding that of 6 children with aFVIII, all had positive LA on at least one visit [162] (see later on LA). One may suspect that the reason for excluding aFVIII from lists of aPL is not because of definition of aPL, but is because it is associated with bleeding, not thrombosis.

#### Conclusion

This section has called attention to the large number of aPL now implicated in APS. It is likely that many more await identification. Second, it illustrates some inconsistencies among reports, discussed later. Third, it appears that the definition or concept of aPL has been guided by the putative association of aPL with thrombosis (APS). Although that is a legitimate concern in view of much evidence, we must not be blinded to the likelihood that some aPL may be involved with very different pathologies, such as MS or ITP, as discussed later.

### Hypotheses for aPL-mediated thrombosis

#### Introduction

Many hypotheses purporting to explain why aPL are often associated with thrombosis or recurrent fetal loss have been tendered. Some were already indicated, such as interference with protein C system. This section briefly reviews other prominent hypotheses, as well as some which are less well known, such as anti-idiotype network dysregulation. Additional theories are considered in connection with LA, discussed later. Space limitations preclude discussion of details of mechanisms, which are found in the references.

#### Platelet and/or endothelial activation

Probably the most widely held hypothesis is that some aPL may activate cells to promote thrombosis. This was well articulated by Vermylen et al [17] and Arnout [139] who proposed a two-hit scenario: an initial weak or subclinical activation, such as of platelets, exposing sufficient anionic PL to favor binding of β_2_GPI or anti-β_2_GPI or other aPL, followed by full-blown thrombotic activation, possibly involving Fc receptors. Related hypotheses had been advanced [163-166]. Specific cross-reaction of aPL with platelet-specific antigens has been reported [167].

Many have proposed a central role for endothelial cells (EC) as targets of aPL [168-175]. There is little doubt that the endothelium is centrally involved in APS [176-178]. E-selectin (CD62E) may be a key player in EC activation [179,180]. Induction of tissue factor (TF) expression in EC by aPL binding could initiate thrombosis in APS [181,182].

Our group has reported that chronic platelet activation, not endothelial activation, distinguishes aPL+ subjects with history of thrombosis from those without such history, by two independent measures (p = 0.003, p = 0.001) [183].

Among the evidences cited by Arnout for initial small damage being amplified by aPL is that recurrence of APS tends to affect the same site or vasculature, suggesting that the site of initial injury is repeatedly targeted. The site of a pinch injury in mice becomes the site of thrombosis in an APS animal model [139].

Dueymes et al cites several models of aPL-EC interaction [184]. Many studies of anti-EC (aEC) independent of aPL have been published [185-188] but others have considered aEC in specific relation to aPL [189-193]. These reports suggest that there is no sharp demarcation between aPL and aEC or even anti-platelet Ab, since many PL-binding protein targets are shared by multiple hematopoietic cells, and the surface PL exposed are similar in the activated state. Heterogeneity of both aEC and aPL has been observed [194].

#### Role of complement (C)

The possible role of C in APS is often mentioned in passing, but a recent review gives more prominence to it [22], as has Shoenfeld [195]. Fischetti et al, working with an animal model of APS sensitized with lipopolysaccharide (LPS) gave persuasive support for a central role of C [196]. Munakata et al showed that C-fixing aCL are specifically associated with thrombotic events [197]. The role of C may be particularly important in APS with cerebral ischemia [198]. In general, IgM is more potent than IgG in fixing complement but this rule varies with subclass, IgG1 = IgG3 > IgG2 > IgG4, and seems to depend on the hinge region [199]. Thus, IgG subclass could be a determinant of thrombogenicity by C-fixing aPL.

Hinton suggested that aCL could be a secondary response to antigens exposed as a result of initial tissue injury [200]. Antibodies to mitochondria, which are rich in CL and are not normally exposed, were recently reported [201]. It was shown in the 1970s that exposure of heart mitochondria caused C activation [202,203]. Later, Kagiyama et al [204] demonstrated that heart mitochondrial molecules bind and fix C even in absence of IgG, although normal human plasma contains C-fixing antibodies that also react. A review of aPL in CAD notes a possible role of C and cites work by Davis and Brey suggesting a role of C in stroke [205]. To the extent that APS is C-dependent, the newly available C-inhibiting drugs may be more effective than anticoagulation alone.

#### Recurrent fetal loss (RFL) and complement

RFL is among the diagnostic criteria of APS. Close associations between RFL and specific aPL have been reported, some of which were mentioned earlier: aAnV [97,121,122], IgA aCL [206], aFXII [161], anti-mitochondria [201], aCD36 [207], anti-β_2_GPI [208,209] and aCL or protein S deficiency [210]. Shoenfeld and colleagues exposed rat embryos and placental explants to IgG from women with RFL, and convincingly demonstrated adverse effects of CL-dependent anti-β_2_GPI specifically [211]. Recently, Salmon and colleagues have made what appears a decisive advance [212]. Using IgG from women with RFL in a mouse model, they demonstrated an absolute requirement of C activation for RFL [212]. She concludes that RFL is an inflammatory condition and that C inhibitors may be the preferred therapy, pointing out that the efficacy of heparin in APS may rely on its C inhibitory action. Thus, it is possible that the unifying feature of the many aPL which have been linked to RFL is the propensity of a given aPL to fix and activate C, particularly in an inflammatory setting. Furthermore, C in conjunction with specific Ab can elicit a wide array of clinical features, and therefore may be a common denominator in aPL disorders.

#### Role of cell-derived microparticles (MP)

Zwaal et al suggested that circulating platelet-derived MP (PMP), which often express phosphatidylserine (PS), could be players in APS by binding β_2_GPI or PT to expose cryptic epitopes for auto-Ab ("neo-autoantigens") [213]. That scenario implies that the platelets are already activated sufficiently to produce PMP, perhaps the first hit of a two-hit scenario. Nomura et al showed that some aPL bind to PMP [214], which is not unexpected since they express PS. Combes et al later reported on endothelial MP (EMP) associated with LA [215], and Dignat-George has further explored the relation of aPL to MP [216]. Vallar et al reported interaction of β_2_GPI with PMP [217]. Our laboratory has extensively studied MP from various cell types and has reviewed PMP and EMP [218,219].

Some authors have been skeptical of the real importance of MP in thrombosis. However, following work by Hron et al [220] showing that plasma of patients at risk for thrombosis have increased thrombin generation, we demonstrated that the increased thrombin generation seen in those patients resides entirely in the MP fraction [221].

#### Dysregulation of anti-idiotype network

Cheng et al found that normal sera became positive for aCL after heating [222], and this has been repeatedly confirmed [223,224]. Matsuda et al [225] were unable to replicate results of Chen et al, but McIntyre et al showed that was caused by the presence of β_2_GPI in the calf serum diluent [226], and proposed that all normal subjects have aPL but normally masked. Cabiedes et al did further investigations [227]. Kra-Oz et al showed that normal IgG when purified became aCL+ unless mixed back with normal sera [228]. Thus, regardless of explanation, it is clear that many aPL are naturally present but invisible to assays due to an inhibitor of some kind.

Natural Ab are those normally present and are not masked if the antigen is not normally present, as in the case of ABO blood group Abs and others, e.g. [229]. If the antigen is normally present, the natural autoAb is suppressed, either by circulating as an immune complex [230] or by an Ab against the natural Ab, called an anti-idiotype. The anti-nuclear Ab common in SLE and other autoimmune disorders are considered to be natural Ab regulated by anti-idiotypes [231]. The anti-idiotype is usually a polyspecific IgM [232,233]. However, a variety of interpretations exist.

Natural Ab are important in immune defense [234] and dysregulation of the normal anti-idiotype network could explain the emergence of positive aPL in pathological states. Stahl et al isolated warm-type autoAb causing autoimmune hemolytic anemia (AIHA) from plasma and RBC eluates of normal subjects, but the IgM pattern was different between controls and patients, leading them to propose that dysregulation of IgM anti-idiotypes causes the disease [235]. Relatedly, Cabiedes et al detected an aPC natural autoAb with hemolytic activity in normal subjects [230]. Moreau et al demonstrated that anti-FVIII is present in all normal subjects [236].

Pan et al showed that normal controls contain SLE-specific autoAbs which are normally masked [237]. In HIV, dysregulation of anti-idiotypes has been proposed for the aPL patterns seen [238]. Interestingly, SLE patients seem to be protected from HIV/AIDS because they can make an aCL which neutralizes the virus, deleted in normal subjects as autoreactive [46]. Shoenfeld et al has nicely reviewed the principles involved [231,239].

A leading hypothesis for the efficacy of intravenous (i.v.) IgG for APS and other autoimmune diseases is correction of disrupted anti-idiotype networks. Fischer et al, in an effort to explain the benefit of i.v. IgG for ITP, found anti-platelet Ab in the plasma fraction which bound to i.v. IgG and differed from normal controls, indicating a role of anti-idiotypes [240]. Yang et al gave evidence for enhancing Ab in ITP, namely, anti-idiotypes which bind the Fc portion of the anti-platelet Ab, and proposed this to account for the difficulty of detecting anti-platelet Ab in ITP [241]. Three ITP patients who were negative for anti-platelet antibodies became positive after treatment with protein A absorption, and the column eluate was also positive, suggesting that the column treatment caused separation from a masking anti-idiotype. Thus, mounting evidence suggests that dysregulation of natural Ab or anti-idiotype networks may be pivotal to the expression of aPL.

#### Other hypotheses

Several other theories have been advanced. McIntyre et al proposed a pivotal role for anti-kininogen aPL [31]. Yasuda et al have demonstrated that monoclonal aCL markedly inhibits fibrinolysis, as does IgG from APS patients, and proposed a cogent scenario in which impaired fibrinolysis is critical to thrombosis in APS [242]. Others have also implicated defective fibrinolysis, as recently reviewed [243].

#### Conclusion

Each of the theories for the putative prothrombotic action of aPL appears persuasive, and animal studies generally support them. However, as remarked in a review of theories based on anti-β_2_GPI [22], it seems unlikely that they can all be right. It is ironic that APS was discovered largely by the aCL test but the clinical value of this test is now considered marginal, as is anti-β_2_GPI [70,122]. Nevertheless, anti-β_2_GPI remains the focus of efforts to understand thrombosis in APS. An alternative viewpoint, increasingly expressed, is inspired by the multiplicity of target antigens and clinical presentations: all of the theories may be right but each may apply only to a particular constellation of antibodies in a given patient. In this view, we are faced not with a single disorder, but with a broad spectrum of autoimmune conditions, not necessarily all thrombogenic.

### Methodological pitfalls in aPL testing by ELISA techniques

#### Introduction

It is well recognized that conflicting reports are common in the literature on aPL detected by ELISA methods. Several examples were given in the foregoing survey of aPL antigens, such as the variable clinical significance of aPT assay depending on method [106]. Reported prevalence of aPL in ITP and MS range from negligible to nearly 90% positive. Reported correlations between a specific pathology and any given aPL frequently vary from insignificant to highly significant. A major cause of these discrepancies is variables in methods. Many reviews of aPL call attention to this problem [21,43,244], that of McIntyre et al being notably detailed [42]. A related problem is frequent failure to describe details of methods in published reports. The following comments bring attention to the most easily overlooked pitfalls. The conclusion to this section further explains why this topic is so important.

#### Plate plastic

Many conflicting reports on anti-β_2_GPI were cleared up when it was discovered that β_2_GPI binds in reactive conformation only to polystyrene ELISA plates that have been treated by gamma radiation [26,245,246]. This was the case also for aPT [105]. According to McIntyre [42], the organic solvents used to dissolve PL may also affect the binding properties. Some authors apply PL from an aqueous suspension of sonic-exposed PL [247]. Such details can spell the difference between positive and negative result, but are not always reported.

#### Source and handling of PL

McIntyre et al compared PE from 6 sources and found significant differences in assay results [42]; see also [248]. Several studies have shown that positivity for a particular protein cofactor can depend on whether it is present alone on the plate or in complex with PL. Thus, not only do results differ between β_2_GPI alone *vs. *β_2_GPI/CL, but PT alone on the plate showed no correlation with APS whereas the complex of PT/PS resulted in good correlation with APS (and LA) [106].

Donohoe et al explored variations of aPT methods and found conditions where aPT IgM, but not IgG, significantly associated with thrombosis [107]. Therefore, contrary to many judgments, aPT may indeed have real clinical significance, but only if assayed in certain ways. Atsumi et al [106] showed that the clinical relevance of aPT depends on the method of detection: the complex of aPT with PS (aPT/PS) was associated with thrombosis but aPT itself was not.

Drying of the PL should be done under nitrogen but often is dried in air. McIntyre showed that air causes rapid oxidation of CL to OxCL, and that PS is also prone to oxidation, converting to lyso-PS. Thus, many or most aCL assays actually measure aOxCL, not aCL. Some kits sold as PE in fact provide lyso-PE [42]. Oxidation of protein antigen on the plate can also affect results [85]. The use of PL mixtures, intended to save time by detecting more antibodies in a single test [249] effectively dilutes the amount of each present, reducing sensitivity [42]. Arvieux found that oxidation of β_2_GPI caused either enhanced or decreased binding to Ig: 10 of 20 patients enhanced, 10 decreased [85].

#### Blocking, washing, dilution

Blockers and diluents include gelatin, non-fat milk, polyethylene glycol (PEG), bovine serum albumin (BSA), fetal calf serum (FCS), etc., at various concentrations and pH, with or without EDTA, detergent, and so on. Kilpatrick compared PEG, BSA and FCS, as well as heat inactivation of FCS and the effect of solvents [250]. One study of inter-lab variability listed some of these different practices [251] but clear conclusions could not be reached owing to the many variables.

Ming and Fan showed that the neutral detergent, Tween 20, which was, and still is, widely used in ELISA for aPL to minimize non-specific binding, markedly enhanced the sensitivity of aCL assay [252]. Cabral et al, for reasons they could not explain, were unable to replicate that result, finding the contrary, that Tween 20 reduced or eliminated detection of aCL, and urged against it [253]. However, close reading shows that Ming and Fan used Tween only as a diluent whereas Cabral et al used it to wash the dried CL three times, probably dissolving most of the CL. Another report found the effect of Tween 20 useful for distinguishing aCL that is dependent *vs*. independent of β_2_GPI [254]. Tween is commonly used at concentrations over a ten-fold range (0.01% to 0.1%), e.g. [251].

#### Heat inactivation; temperature

Heat inactivation of the serum caused a drop in apparent aCL titer [250,255]. Most assays are run at room temperature but some use 37°C. It has been shown that temperature markedly affects binding of IgG *vs*. IgM [256]. Heating can inactivate some complement components and, as earlier mentioned, can unmask some natural antibodies.

#### Calcium

Many PL-binding proteins require Ca^2+ ^to bind, yet Ca^2+ ^is rarely present in assays. It has been shown that Ca^2+ ^is an absolute requirement for assay of aPT [105] but methods vary. Of two aFVII purified from a patient, one depended on Ca^2+^, the other did not [257]. β_2_GPI binds to PL in a calcium-dependent fashion despite absence of gla domains [217], but another study found that Ca^2+ ^(2 mM) reduced binding to platelet microparticles [217]. Conversely, use of EDTA could affect results by extracting endogenous Ca^2+^, thereby affecting epitope conformation of some proteins.

#### Serum *vs *plasma

Plasma contains many coagulation factors and other agents while serum does not, and conversely, serum contains Ca^2+ ^and active proteolytic enzymes. Therefore, results could differ, depending on the agent tested for. Wong et al found no difference between plasma and serum for anti-β_2_GPI/CL [258] (and pointed out a common error in statistics). We recently demonstrated gross differences in assay of CD40L in plasma *vs. *serum [259], leading to suspicion of similar effects for at least some aPL. Freeze-thaw cycles of the sample may also affect status of protein-lipid complexes, free proteins, immune complex, or aggregated IgG.

#### Calculations: lesson from IgA

Results can differ depending on the cutoff defining a positive test, e.g. 1, 2, or 3 SD above normal mean, or more (4 SD [123], 6 SD [125]). The definition of blank to be subtracted can be even more important, as illustrated by some reports on IgA aPL. A large study found only 2 IgA+ aCL among 795 patients [260]. Close reading, however, reveals that they blocked the CL-coated plates with FCS and then subtracted the result with FCS alone (no CL), calling this "non-specific binding" (NSB), proposed as an important refinement. But the data showed that NSB varied widely, which is unexpected for true NSB, leading to the more likely conclusion that many of the sera were reacting to a component of the FCS. In contrast, Baleva et al in the following year [206] found significant IgA aCL in several patient groups, and in some groups, more IgA than IgG. Indeed, the authors note that IgA was *the only *Ig detected in 8 cases, and cite references compatible with their findings. A study of aPL in diabetes found that IgA aPL reacting to PE was more frequent than IgG or IgM [261]. Thus, one may question the conclusion, widely repeated, that IgA testing is not useful.

#### Conclusion

Methodological differences almost certainly accounts for the majority of conflicting reports. This is important because deciding the clinical value of a given test, such as aCL or anti-β_2_GPI, depends on which reports are believed. Careful study of methods may reveal the explanation for discrepancies. For the same reason, one must be suspicious of meta-analyses which, in effect, average together a large number of reports, thereby nullifying those which are favorable. Galli et al acknowledge this point in the concluding sentence of their abstract [244], and others have commented similarly [262,263]. To address this problem, many workshops aimed at standardizing methods have been held [251,264,265], as recently discussed [266]. Standard methods are clearly mandatory for clinical testing, although problems have persisted [248,267]. However, this could be counter-productive since variant methods may give more favorable results for a particular antigen or clinical condition. For research purposes, important discoveries could critically depend on variant methods, as illustrated by some of the examples cited. Future technology may enable dozens of assays per patient, easily and cheaply, by robotic arrays.

### The lupus anticoagulant (LA)

#### Background: The LA paradox

As its name implies, the LA acts *in vitro *like an anticoagulant, prolonging the activated partial thromboplastin time (APTT) or other tests specifically designed to detect it. According to a 1961 review [268], this effect was first reported in 1946 in a patient with ITP [269]. Its frequent association with SLE was reported in a series of papers by Conley and colleagues from 1948 [270] to 1952 [2]. Both papers cited describe LA in the setting of hemorrhagic diatheses, as expected of an anticoagulant. The latter is usually cited as the origin of the LA test, although the name, LA, was not coined until 1972.

However, Bowie et al in 1963 described four cases of SLE with thrombosis despite presence of LA [271]. This was the first clear statement of the paradox: *why should thrombosis occur in the presence of an anticoagulant? *Efforts to answer this persist to the present.

Subsequently, a growing number of reports through the 1970s found association of LA with thrombosis at greater frequency than hemorrhage, culminating with recognition of the aPL syndrome (APS) in the mid-1980s, as earlier referenced [3-5,272].

#### Principles of LA testing

Although the LA is widely spoken of as an aPL, it must be stressed that LA is not defined by any specific antibody or other known agent, but only by its effect in an LA assay. In its simplest early form, the LA assay was the recalcification time (recal time) of platelet-poor plasma (PPP), together with the "platelet neutralization test" (PNT) for confirmation [268]. Briefly, one adds sufficient calcium to overcome the citrate in the PPP and observes if the time to coagulation is abnormally long, indicating LA+, provided that in addition, the prolongation is reduced by the PNT (originally, addition of platelets or platelet membranes). This was interpreted to mean that some factor in the plasma, called the LA, was acting to block sites on the phospholipids (PL) essential for coagulation, suggesting that aPL is that factor. The rationality of the PNT is that an excess of PL overwhelms the ability of the LA to block enough sites to prolong the time. A variety of LA tests are now in use, beyond the scope of this review, but all are similar in principle.

#### LA associates best with thrombosis

Among the first persuasive reports of a strong association between LA and thrombosis was the retrospective analysis of Mueh at al in 1980 [273]. Following recognition of the syndrome, APS, it became common to test LA in parallel with solid-phase ELISA of aPL, leading to several reports showing that LA has a stronger association with thrombosis than aPL measured by ELISA, notably by Derkens et al in 1988 [274], later confirmed repeatedly [6,275-277], explicitly stated by Horbach et al in 1996 [278] and by Arnout in 2001 [11] and, if further confirmation was needed, in a meta-analysis by Galli in 2003 [244]. Indeed, several studies found that thrombosis associated closely with LA but poorly or not at all with aPL by ELISA, e.g. [276,277].

Thus, it is firmly established that the association of LA+ assay with thrombophilic states is much stronger than ELISA of aPL such as aCL, anti-β_2_GPI, or other. Furthermore, this association is robust with respect to variant methods. However, if it is true that LA is an aPL, we are faced with the problem of explaining why LA correlates so much better with thrombosis than aPL measured by ELISA.

#### What is the LA? Why thrombogenic?

As explained by Triplett [7], early results of Harris et al [3] suggested to them that LA and aCL were one and the same entity. This was later shown to be incorrect since the two were clearly separable. Nevertheless, the assumption continued that LA is a manifestation of aPL, to the point where LA is spoken of almost as a synonym for aPL, or as a particular type of aPL [279]. Indeed, Triplett has stated that LA is an aPL, by definition [7].

As detailed in several reviews, such as by Arnout [11], it has been shown that at least some LA are in fact expressions of anti-β_2_GPI. Roubey et al showed that LA plasma added to normal plasma prolonged coagulation but not if the plasma was first depleted of β_2_GPI [279]. Around the same time, Arvieux showed that mouse antibodies against β_2_GPI had LA-like activity [280]. Arnout made a series of monoclonal antibodies (mAb) against β_2_GPI and found that only 7 of 21 mAb had LA activity, variable in degree, one being very strong [28,281]. This suggested that the epitope targeted was critical for LA activity.

According to Arnout [11], the now accepted explanation for the LA activity of anti-β_2_GPI was developed by three groups, Willems at al in 1996 [282]), Takeya et al in 1997 [283], and Arnout et al in 1998 [28]. The essence of it is that only those anti-β_2_GPI which can bind two molecules of β_2_GPI (divalently) in the soluble phase cause enhanced binding to PL and exhibit LA activity [11]. However, not all LA depend on β_2_GPI.

In the same timeframe, others had shown that aPT can also exert LA activity, notably Bevers et al and Galli et al in 1992 [284], Oosting et al in 1993 [72], and Permpikul et al in 1994 [285], as referenced [7,11]. A method was devised to discriminate between LA that depended on PT from LA that depended on β_2_GPI [286]. The explanation given for why some but not all aPT exhibit LA activity is similar to that given for anti-β_2_GPI. This explanation does not, however, directly account for thrombosis, except by way of the hypotheses listed above for aPL in general.

Field et al [287,288] in their introduction give references to about five different theories for LA but dismiss them all as unconvincing, and then supply evidence for a novel explanation of their own: IgG from LA plasma inhibits thrombin generation only under static conditions, whereas under the shearing conditions of natural blood flow, LA promotes thrombin generation. In that scenario, the LA effect is an *in vitro *artifact.

Several workers have proposed anti-annexin V (aAnV) as the agent of LA, with direct bearing on thrombosis. Matsuda et al found that aAnV was common in SLE patients (positive in 12 of 47) and associated strongly with LA activity [123]; for commentary, see [124]. The LA activity of aAnV was further explored by Nakamura et al the next year [90]. It is possible that aAnV may also bear on the antagonism between annexins and phospholipase A_2 _(PLA2) [126].

In a series of papers, Rand et al has proposed to resolve the LA paradox on the basis of competition between aPL and the natural anticoagulant function of AnV [289-291]. They showed that aPL IgG inhibits the anticoagulant effect of exogenously added AnV. However, the strong action of added AnV is contrary to the hypothesis that AnV is naturally present in plasma in significant amounts. Although AnV exhibits an effect on coagulation indistinguishable from LA [112], it has not been shown to play a significant role as a natural anticoagulant, except perhaps in pregnancy.

Most reviewers now accept the existence of multiple LA, not necessarily limited to anti-β_2_GPI, aPT and/or aAnV. For example, anti-FVIII behaves like LA [292]. A study of LA+ patients found that all of them reacted to at least one pure PL, and 95% were positive for aPE [146]. The specific antigen(s) was not identified. We have speculated that TFPI (the inhibitor of tissue factor) could exhibit LA activity, since it prolongs coagulation, could be neutralized by excess PL, and in at least some circumstances, is elevated in thrombophilic state [293]. Thus, quite different explanations are conceivable.

#### Conclusion

Although it has been clearly shown that some aPL exhibit LA activity, and have pathological effects in animal models, the specific identity of the LA relevant to thrombosis is unknown in many cases. Moreover, why LA associates so much better with thrombosis compared to aPL test by ELISA remains an open question. As deLaat et al wrote in 2007, the association of LA with thrombosis is "for yet unknown reasons" [291].

### Antiphospholipid antibodies (aPL) in non-APS, non-SLE disorders

It has long been recognized that aPL occur at high frequency in many disorders other than APS and SLE, especially those known to be immune-mediated, such as immune thrombocytopenic purport (ITP), multiple sclerosis (MS), and rheumatoid arthritis (RA). However, the significance of aPL in these disorders has been generally dismissed as non-specific or epiphenomenal, partly because the aPL did not appear to be related to symptoms, and perhaps also because aPL in these disorders is inconsistent with the paradigm that the pathological significance of aPL is limited to thrombosis. This review was motivated in part by recent findings which indicate that aPL are in fact associated with symptoms in non-APS, non-SLE disorders in humans.

#### Multiple sclerosis (MS)

MS is an inflammatory disorder believed to be autoimmune in etiology, and can present with features resembling APS [294-297]. Several reviews of the neurological symptoms of APS/SLE are available [298-300], and many case reports, e.g., cerebral ischemia [301]. However, MS is not thought to involve ischemia, although elements of the coagulation cascade are present in MS lesions, including fibrinogen and recently, tissue factor and protein C inhibitor [302].

In 2000, our collaborative investigation demonstrated elevated endothelial microparticles (EMP) during exacerbations of MS [303,304]. Those findings motivated further investigations, this time of aPL in MS, with the hypothesis that aPL might be involved in endothelial activation in MS. Several prior reports had established that aPL commonly occur in MS, but in most of them the patient population was heterogeneous or inadequately defined, and there was no indication of a relation between aPL and the pathophysiology of MS.

To examine the relationship more closely, we tested samples of well-defined, treatment-naive MS patients in either exacerbation or remission, documented by neurological as well as brain MRI with and without contrast. The central finding was that all aPL measured were significantly elevated in acute phases *vs. *remission, and correlated strongly with MRI imaging, p = 0.002 [136]. The antigens tested included β_2_GPI, FVII, and four pure PL (CL, PC, PS, PE). Of interest, aFVII was never detected in remission but was present in 60% of acute MS; and anti-β_2_GPI was positive in 80% of acute MS. It is possible that unidentified and possibly MS-specific auto antibodies were also present, judging by the strong reaction to the pure PL in acute, but not remission, cases. Unexpectedly, aPL in MS were exclusively of IgM class, with no IgG detected.

Because that work showed a direct relation between aPL and clinical state in MS, it is plausible to suspect that aPL may be involved in the pathogenesis of MS. Of course, the possibility exists that aPL in acute MS are epiphenomenal; but the same argument could be levelled against the hypothesis that aPL cause thrombosis. In further support, Shoenfeld and colleagues clearly demonstrated neuropathological effects of aPL in animal models [305-307].

Since some aPL have been identified with anti-endothelial (anti-EC) antibodies (earlier cited), and since our group [303] and others have documented endothelial activation in MS, it is relevant to note that anti-EC have been detected in MS and were proposed to contribute to its pathogenesis. In 1989, Tsukada et al. found anti-EC in 75% of active MS but in only 4% of remission [308]. However, a 1992 report found only 13% positive [309] and a later report found only 10% reactive to human umbilical vein EC (HUVEC) [310]. On the other hand, another report around the same time, but using brain microvascular EC rather than HUVEC, found that 12/16 active vs 0/15 inactive MS reacted to EC [311]. This suggests that anti-EC in MS are specific for brain microvessels, and would be consistent with the fact that CNS lesions in MS tend to develop around brain microvessels (Dawson fingers) [312]. Thus, brain-specific anti-EC could be a pivotal pathogenic mechanism of aPL in MS.

#### Immune thrombocytopenic purpura (ITP)

As with MS, it was long known that aPL commonly occur in ITP, but no one had related their presence to clinical state (low platelets, bleeding), therefore their presence was dismissed as inconsequential. Y.S. Ahn, a specialist in ITP, had drawn attention to cerebral ischemia sometimes associated with ITP, particularly in splenectomized patients, leading to vascular dementia [313].

To investigate if aPL might be involved, we studied series of patients stratified by clinical state (acute, chronic, remission) and demonstrated, for the first time, a clear association between elevated aPL and onset of acute symptoms [314]. Sequential study of six cases confirmed the general conclusion, that aPL rise with exacerbations and decline or disappear in remission of ITP. Unexpectedly, however, no relation was detected between aPL/LA and those patients with ITP-associated cerebral ischemia.

In a follow-up study, Bidot and colleagues [315] compared aPL patterns in APS *vs. *active ITP (remission excluded). One notable difference was that LA was absent in all ITP patients but was present in 24/33 (79%) of APS. This supports the unique significance of LA in thrombosis. IgG anti-β_2_GPI was >3-fold more common in APS than ITP, but positive reaction to the pure PL (CL, PC, PS, PE) was more common in ITP, p < 0.05. This need not imply that aPL in ITP are mainly of the so-called non-pathogenic type, since it is possible that the pure PL are reacting to unidentified antigens in the plasma, as they would to platelet membranes.

Since CD36 is often found in association with aPL, it is apropos to mention our findings on CD36 in ITP. On platelets, this antigen is known as glycoprotein IV (GpIV), and was of interest to us in thrombotic thrombocytopenic purpura (TTP) [316] and other thrombosis [317]. More recently, we found that aCD36 (and some other anti-platelet autoantibodies) is more commonly elevated in ITP patients with bleeding symptoms than in comparable patients without bleeding (unpublished), unexpected since aCD36 is usually associated with thrombosis, as earlier mentioned.

#### HIV/AIDS

A viral cause of APS has been proposed [318]. HIV infection carries a high frequency of aPL [34,319] but here, too, the aPL in HIV were considered to be of the infection-related type and non-pathogenic. Haynes et al [46] point out that anti-HIV antibodies mounted by most patients fail to neutralize the virus, but a rare few do mount neutralizing responses, and those studied turned out to be polyspecific aCL, similar to the aPL profile seen in lupus (SLE). Indeed, they cite references indicating that SLE patients appear to be protected against contracting HIV, and argue that the general population fails to make such aPL because they have been deleted from the repertoire as self-reactive. In support of their contention, one of the neutralizing anti-HIV they studied was autoreactive with dsDNA, centromere B, histones and other self targets [46]. Relatedly, Zhang et al [320] investigated why most people fail to mount effective immune responses to HIV envelope proteins (Env), and suggested that Env suppresses CD40L expression, which in turn blunts the T cell ability to activate DCs. However, we feel that findings of Zhang et al [320] are consistent with the scenario given by Haynes et al [46]. Specifically, the aPL seen in the context of HIV and other infections may be more than epiphenomena and could offer important clues to immune function.

## Conclusion

The thrust of this review has been to highlight some of the uncertainties and challenges in the field. To begin with, the proliferation of target antigens over the last 20 years has greatly broadened the classical concept of "aPL," and calls into question the definition of aPL. The strength of the association of aPL (measured by ELISA) with thrombosis has been questioned, but this depends on which reports are believed. On the other hand, there is no doubting the close association of LA with thrombosis; but exactly why this is true remains unsettled. Finally, new evidence is presented indicating that aPL may be involved with the pathogenesis of other disorders, notably MS and ITP, as distinct from the role of aPL exclusively in thrombosis.

The picture now emerging is that aPL are part of a large spectrum of autoantibodies, including, for example, those of ITP, and that APS is just one manifestation of a particular constellation of aPL. We may be better served by abandoning the concept that aPL are exclusively thrombogenic.

In regard to the cause of aPL-associated pathologies, a promising hypothesis is dysregulation of anti-idiotype networks. Many of the consequences appear to be best explained in terms of complement-mediated effects. However, full understanding of the aPL phenomenon remains a challenge for the future.

## Competing interests

The authors declare thtat they have no competing interests.

## Authors' contributions

LLH, WJ, YSA, REK, CJB, AHM, RZ, ME, AHM, SAM, and AM performed extensive literature research, prepared the manuscript and provide expertise in interpretation of data obtained from several sources. REK, RZ, AHM, SAM, AM, and ME reviewed the manuscript extensively and provided constructive comments to improve the quality of the manuscript.

CB performed the actual aPL assays, was first author of key papers cited, and commented helpfully on versions of the manuscript.

LLH, WJ, YSA, REK, RZ, and AM provided clinical expertise in various fields of neuroinflammation and improved the quality of the original manuscript.

All authors worked as team members to generate this extensive review.

## References

[B1] Eng A (1994). Cutaneous expression of antiphospholipid syndromes. Sem Thomb Haemost.

[B2] Conley CL, Hartman RC (1952). A hemorrhagic disorder caused by circulating anticoagulant in patients with disseminated lupus erythematosus. J Clin Invest.

[B3] Harris EN, Charavi AE, Boey ML, Patel BM, Mackworth-Young CG, Loizou S, Hughes GRV (1983). Anticardiolipin antibodies: detection by radioimmunoassay and association with thrombosis in systemic lupus erythematosus. Lancet.

[B4] Harris EN, Charavi AE, Hedge U, Derue G, Morgan SH, Englert H, Chan JKH, Asherson RA, Hughes GRV (1985). Anticardiolipin antibodies in autoimmune thrombocytopenic purpura. Brit J Haematol.

[B5] Hughes GRV, Harris EN, Gharavi AE (1985). The anticardiolipin syndrome. J Rheumatol.

[B6] Ginsberg JS, Wells PS, Brill-Edwards P, Donovan D, Moffatt K, Johnston M, Stevens P, Hirsh J (1995). Antiphospholipid antibodies and venous thromboembolism. Blood.

[B7] Triplett DR (1995). Protean clinical presentations of antiphospholipid antibodies (APA). Thromb Haemost.

[B8] Galli M, Barbui T (1999). Antiprothrombin antibodies: detection and clinical significance in the antiphospholipid syndrome. Blood.

[B9] Wilson WA, Gharavi AE, Koike T, Lockshin MD, Branch DW, Piette JC, Brey R, Derksen R, Harris EN, Hughes GRV, Triplett DA, Khamashta MA (1999). International consensus statement on preliminary classification criteria for definite antiphospholipid syndrome. Arthritis Rheum.

[B10] Lockshin MD, Sammaritano LR, Schwartzman S (2000). Validation of the Sapporo criteria for antiphospholipid syndrome. Arthritis Rheum.

[B11] Arnout J (2001). Antiphospholipid syndrome: diagnostic aspects of lupus anticoagulants. Thromb Haemost.

[B12] Rand JH, Macik BG, Konkle BA, Schecter GP, Broudy VC, Williams ME, Bajus JL (2001). Thrombophilia: What's a practitioner to do? (Diagnosis and treatment of the antiphospholipid syndrome).

[B13] Levine JS, Branch DW, Rauch J (2002). The antiphospholipid syndrome (Review). N Engl J Med.

[B14] Brey RL, Levine SR, Stallworth CL, Asherson RA, Cervera R, Piette JC, Shoenfeld Y (2002). Neurologic manifestations in the antiphospholipid syndrome. The Antiphospholipid Syndrome II: Autoimmune Thrombosis.

[B15] Hughes GRV (1993). The antiphospholipid syndrome: ten years on. Lancet.

[B16] Roubey RAS (1996). Immunology of the antiphospholipid antibody syndrome. Arthritis Rheum.

[B17] Vermylen J, Hoylaerts MF, Arnout J (1997). Antibody-mediated thrombosis. Thromb Haemost.

[B18] Galli M, Finazzi G, Barbui T (1997). Antiphospholipid antibodies: predictive value of laboratory tests. Thromb Haemost.

[B19] Esmon NL, Smirnov MD, Esmon CT (1997). Thrombogenic mechanisms of antiphospholipid antibodies (State of the Art). Thromb Haemost.

[B20] Greaves M (1999). Antiphospholipid antibodies and thrombosis [for comments, see v354:71-3]. Lancet.

[B21] Arnout J, Vermylen J (2003). Current status and implications of autoimmune antiphospholipid antibodies in relation to thrombotic disease. J Thromb Haemost.

[B22] Giannakopoulos B, Passam F, Rahgozar S, Krilis SA (2007). Curent concepts on the pathogenesis of the antiphospholipid syndrome. Blood.

[B23] Matsuura E, Igarashi Y, Fujimoto M, Ichikawa K, Koike T (1990). Anticardiolipin cofactor(s) and differential diagnosis of autoimmune diseases. Lancet.

[B24] Galli M, Comfurius P, Maassen C, Hemker HC, DeBaets MH, vanBreda-Vriesman TJC, Barbui T, Zwaal RFA, Bevers EM (1990). Anticardiolipin antibodies directed not to cardiolipin but to a plasma cofactor. Lancet.

[B25] McNeil HP, Simpson RJ, Chesterman CN, Krillis SA (1990). Anti-phospholipid antibodies are directed against a complex antigen that includes a lipid-binding inhibitor of coagulation: beta 2 glycoprotein 1 (apolipoprotein H). Proc Nat Acad Sci USA.

[B26] Ichikawa K, Khamashta MA, Koike T, Matsuura E, Hughes GRV (1994). β2-glycoprotein 1 reactivity of monoclonal antibodies from patients with the antiphospholipid syndrome. Arthritis Rheum.

[B27] Forastiero RR, Martinuzzo ME, Kordich LC, Carreras LO (1996). Reactivity to beta 2 glycoprotein 1 clearly differentiates anticardiolipin antibodies from antiphospholipid syndrome and syphilis. Thromb Haemost.

[B28] Arnout J, Wittevrongel C, Vanrusselt M, Hoylaerts M, Vermylen J (1998). Beta-2-glycoprotein 1 dependent lupus anticoagulants form stable bivalent antibody beta-2-glycoprotein 1 complexes on phospholipid surfaces. Thromb Haemost.

[B29] Day HM, Thiagarajan P, Ahn C, Reveille JD, Tinker KF, Arnett FC (1999). Autoantibodies to β2-glycoprotein 1 in systemic lupus erythematosus and primary antiphospholipid antibody syndrome: clinical correlations in comparison with other antiphospholipid antibody tests. J Rheumatol.

[B30] Arvieux J, Roussel B, Jacob MC, Colomb MG (1991). Measurement of anti-phospholipid antibodies by ELISA using β2-glycoprotein 1 as an antigen. J Immunol Meth.

[B31] McIntyre JA, Wagenknecht DR, Sugi T (1997). Phospholipid binding plasma proteins required for antiphospholipid antibody detection: an overview. Am J Reprod Immunol.

[B32] Roubey RA (1994). Autoantibodies to phospholipid binding proteins: a new view of lupus anticoagulants and other 'antiphospholipid' autoantibodies. Blood.

[B33] Schultz DR (1997). Antiphospholipid antibodies: basic immunology and assays. Sem Arthritis Rheum.

[B34] Petrovas C, Vlachoyoannopoulos PG, Kordossis T, Moutsopoulos HM (1999). Anti-phospholipid antibodies in HIV infection and SLE with or without anti-phospholipid syndrome: comparisons of phospholipid specificity, avidity and reactivity with beta2-GP1. J Autoimm.

[B35] Loizou S, Singh S, Wypkema E, Asherson RA (2003). Anticardiolipin, anti-beta2-glycoprotein 1 and antiprothrombin antibodies in black South African patients with infectious diseases. Ann Rheum Dis.

[B36] Chairman, Arnout J (2002). ISTH Scientific Standardization Subcommittee: Lupus Anticoagulants/Phospholipid-Dependent Antibodies. Boston, MA.

[B37] Nash MJ, Camiller RS, Kunka S, Mackie IJ, Machin SJ, Cohen H (2004). The anticardiolipin assay is required for sensitive screening for antiphospholipid antibodies. J Thromb Haemost.

[B38] Meroni PL, Tincani A (2004). Should our approach to the anticardiolipin assay change 20 years after its discovery? [Commentary on Nash, et al, pg 1077]. J Thromb Haemost.

[B39] DeMoerloose P, Reber G (2004). Antiphospholipid antibodies: do we still need to perform anticardiolipin ELISA assays? [Commentary on Nash, et al, pg 1077]. J Thromb Haemost.

[B40] Harris NE, Goldsmith G, Pierangeli S, Gharavi A, Branch W (1995). Phospholipid binding antibodies warrant continued investigation. Blood.

[B41] Pierangeli SS, Harris EN, Gharavi AE, Goldsmith G, Branch DW, Dean WL (1993). Are immunoglobulins with lupus anticoagulant activity specific for phospholipids?. Br J Haematol.

[B42] McIntyre JA, Wagenknecht DR, Faulk WP (2003). Antiphospholipid antibodies: discovery, definitions, detection and disease. Prog Lipid Res.

[B43] Merrill JT (2001). Which antiphospholipid antibody tests are most useful?. Rheum Dis Clin North Am.

[B44] vonLandenberg C, Lackner KJ, vonLandenberg P, Lang B, Schmitz G (1999). Isolation and characterization of two human monoclonal anti-phospholipid IgG from patients with autoimmune disease. J Autoimm.

[B45] Sorice M, Circella A, Griggi T, Garofalo T, Nicodemo G, Pittoni V, Ponteri GM, Lenti L, Valewsini G (1996). Anticardiolipin and anti-β2-GP-I are two distinct populations of autoantibodies. Thromb Haemost.

[B46] Haynes BF, Fleming J, StClair EW, Katinger H, Stiegler G, Kunert R, Robinson J, Scearce RM, Plonk K, Staats HF, Ortel TL, Liao HX, Alam SM (2005). Cardiolipin polyspecific autoreactivity in two broadly neutralizing HIV-1 antibodies. Science.

[B47] Metzger J, vonAndenbeerg P, Keerel M, Buhl A, Lackner KL, Luppa PB (2007). Biosensor analysis of beta-2-glycoprotein I reactive autoantibodies: Evidence for isotype-specific binding and differentiation of pathogenic from infection-induced antibodies. Clin Chem.

[B48] Amengual O, Atsumi T, Khamashta MA, Koike T, Hughes GRV (1996). Specificity of ELISA for antibody to β2-glycoprotein 1 in patients with antiphospholipid syndrome. Br J Rheumatol.

[B49] Amiral J, Larrivaz I, Cluzeau D, Adam M (1994). Standardization of immunoassays for antiphospholipid antibodies with β2 GP1 and role of other phospholipid cofactors. Haemostasis.

[B50] Galli M, Bevers EM, Comfurius P, Barbui T, Zwaal RFA (1993). Effects of antiphospholipid antibodies on procoagulant activity of activated platelets and platelet-derived microvesicles. Br J Haematol.

[B51] Sugi T, McIntyre JA (1996). Phosphatidylethanolamine induces specific conformational changes in the kininogens recognizable by anti-phosphatidylethanolamine antibodies. Thromb Haemost.

[B52] Sugi T, McIntyre JA (2001). Certain autoantibodies to phosphatidylethanolamine (aPE) recognize factor XI and prekallikrein independently or in addition to the kininogens. J Autoimm.

[B53] Tomas JF, Tomas JF, Alberca I, Tabernero MD, Cordero M, DelPino-Montes J, Vicente V (1998). Natural anticoagulant proteins and antiphospholipid antibodies in systemic lupus erythematosus. J Rheumatol.

[B54] Pratico D, Ferro D, Iuliano L, Rokach J, Conti F, Valesini G, FitzGerald GA, Violi F (1999). Ongoing prothrombotic state in patients with antiphospholipid antibodies: A role for increased lipid peroxidation. Blood.

[B55] Pratico D, Tangirala RK, Horkko S, Witztum JL, Palinski W, FitzGerald GA (2001). Circulating autoantibodies to oxidized cardiolipin correlate with isoprostane F2 alpha-VI levels and the extend of atherosclerosis in ApoE-deficient mice: modulation by vitamin E. Blood.

[B56] Horkko S (1997). The epitopes for some antiphospholipid antibodies are adducts of oxidized phospholipid and beta2 glycoprotein 1 (and other proteins). Proc Natl Acad Sci USA.

[B57] Lopes-Virella MF, Virela G, rchard TJ, Koshkinen S, Evans RW, Becker DJ, Forrest KYZ (1999). Antibodies to oxidized LDL and LDL-containing immune complexes as risk factors for coronary artery disease in diabetes mellitus. Clin Immunol.

[B58] Ames PRJ, Nourooz-Zadeh JN, Tommasino C, Alvez J, Brancaccio C, Anggard EE (1998). Oxidative stress in primary antiphospholipid syndrome. Thromb Haemost.

[B59] Amengual O, Atsumi T, Kamashta MA, Hughes GRV (1998). The role of the tissue factor pathway in the hypercoagulable state in patients with the antiphospholipid syndrome. Thromb Haemost.

[B60] Cugno M, Dominguez M, Cabibbe M, Bisiani G, Galli M, Angles-Cano E, Agostoni A (2000). Antibodies to tissue-type plasminogen activator in plasma from patients with primary antiphospholipid syndrome. Br J Haematol.

[B61] Hurtado V, Montes R, Gris JC, Bertolaccini ML, Alonso A, Martinez-Gonzalez MA, Khamashta MA, Fukudome F, Lane DA, Hermida J (2004). Autoantibodies against EPCR are found in antiphospholipid syndrome and are a risk factor for fetal death. Blood.

[B62] vanHylckama VA, Montes R, Rosendaal FR, Hermida J (2007). Autoantibodies against endothelial protein C receptor and the risk of a first deep vein thrombosis. J Thromb Haemost.

[B63] Barrow RT, Healey JF, Jacquemin MG, Saint-Remy JMR, Lollar P (2001). Antigenicity of putative phospholipid membrane-binding residues in factor VIII. Blood.

[B64] Asada Y, Sumiyoshi A, Hayashi T, Syzymiya J, Kaketani K (1985). Immunohistochemistry of vascular lesions in thrombotic thrombocytopenic purpura, with special reference to factor VIII related antigen. Thromb Res.

[B65] Prescott R, Nakai H, Saenko EL, Scharrer I, Nilsson IM, Humphries JE, Hurst D, Bray G, Scandella D (1997). The inhibitor antibody response is more complex in hemophilia A patients than in most nonhemophiliacs with factor VIII autoantibodies. Blood.

[B66] Lollar P (2004). Pathogenic antibodies to coagulation factors. Part I. Factor VIII and IX. J Thromb Haemost.

[B67] Lollar P (2005). Pathogenic antibodies to coagulation factors. Part II. Fibrinogen, prothrombin, thrombin, factor V, factor XI, factor XII, factor XIII, the protein C system and von Willebrand factor. J Thromb Haemost.

[B68] Jones DW, Gallimore MJ, Harris SL, Winter M (1999). Antibodies to factor XII associated with lupus anticoagulant. Thromb Haemost.

[B69] Galli M, Luciani D, Bertolini G, Barbui T (2003). Anti-beta-2-glycoprotein 1, antiprothrombin antibodies, and the risk of thrombosis in the antiphospholipid syndrome. Blood.

[B70] Galli M (2008). Clinical utility of laboratory tests used to identify antiphospholipid antibodies and to diagnose the antiphospholipid syndrome. Sem Thromb Hemost.

[B71] Finazzi G, Brancaccio V, Moia M, Ciavarella N, Mazzucconi MG, Schinco PC, Ruggeri M, Pogliani EM, Gamba G, Rossi E, Baudo F, Manotti C, D'Angelo A, Palareti G, DeStefanon V, Berrettini M, Barbui T (1996). Natural history and risk factors for thrombosis in 360 patients with antiphospholipid antibodies: A four-year prospective study from the Italian Registry. Am J Med.

[B72] Muriel M, Sarzi-Puttini P, Peretti R, Rossi E, Atzeni F, Parsons W, Doria A (2005). Thrombotic risk factors in primary antiphospholipid syndrome: A 5-year prospective study. Stroke.

[B73] Lien LM, Chen WH, Chiu HC, Pan WH, Chen JR, Bai CH (2006). High titers of anticardiolipin antibody is associated with first-ever ischemic stroke in Taiwan. Cerebrovasc Dis.

[B74] McNeeley PA, Dlott JS, Furie RA, Jack RM, Ortell TL, Triplett DA, Victoria EJ, Linnik MD (2001). β2 glycoprotein I-dependant anticardiolipin antibodies preferentially bind the amino terminal domain of β2-glycoprotein 1. Thromb Haemost.

[B75] Kuwana M, Matsuura E, Kobayashi K, Okazaki Y, Kaburaki J, Ikeda Y, Kawakami Y (2005). Binding of β2-glycoprotein I to anionic phospholipids facilitates processing and presentation of a cryptic epitope that activates pathogenic autoreactive T cells [with editorial by Vickers and Greaves, p1371]. Blood.

[B76] deLaat B, Derksen RHWM, Urbanus RT, deGroot PG (2005). IgG antibodies that recognize epitope Gly40-Arg43 in domain I of β2-glycoptorein I cause LAC, and their presence correlates strongly with thrombosis [With editorial by Vickers and Greaves, pg 1371]. Blood.

[B77] Yasuda S, Atsumi T, Ieko M, Kobayashi K, Inagaki J, Kato H, Tanaka H, Yamakada M, Akino M, Saitou M, Aamasaki Y, Jodo S, Amengual O, Koike T (2004). Nicked beta-2-glycoprotein I: a marker of cerebral infarct and a novel role in the negative feedback pathway of extrinsic fibrinolysis. Blood.

[B78] Hulstein JJJ, Lenting PJ, deLaat B, Derksen RHWM, Fijnheer R, deGroot PG (2007). Beta-2-glycoprotein I inhibits von Willebrand factor-dependant platelet adhesion and aggregation. Blood.

[B79] Carron JA, Bates RC, Smith AI, Tetoz T, Arellano A, Gordon DL, Burns GF (1996). Factor H co-purifies with thrombospondin isolated from platelet concentrates. Biochem Biophys Acta, Gene Struct Exp.

[B80] Nimpf J, Bevers EM, Bomans PHH, Till U, Wurm H, Kostner GM, Zwaal RF (1986). Prothrombinase activity of human platelets is inhibited by β2-glycoprotein I. Biochim Biophys Acta.

[B81] Nimpf J, Wurm H, Kostner GM (1987). β2-glyoprotein I (apo-H) inhibits the release reaction of human platelets during ADP-induced aggregation. Atheroscler.

[B82] Schousboe I (1985). β2-glycoprotein I: A plasma inhibitor of the contact activation of the intrinsic blood coagulation pathway. Blood.

[B83] McNally T, Mackie IJ, Isenberg DA, Machin SJ (1996). β2 glycoprotein-1 inhibits factor XII activation on triglyceride rich lipoproteins: The effect of antibodies from plasma of patients with antiphospholipid syndrome. Thromb Haemost.

[B84] Pengo V, Biasiolo A, Brocco T, Rampazzo P (2000). Effect of anti-β2 glycoprotein I lupus anticoagulants on fibrin polymerization and fibrinolysis. Autoimmunity.

[B85] Arvieux J, Regnault V, Hachulla E, Darnige L, Berthou F, Youinou P (2001). Oxidation of β2-glycoprotein 1 (β2GPI) by the hydroxyl radical alters phospholipid binding and modulators recognition by anti-β2GPI autoantibodies. Thromb Haemost.

[B86] Mori T, Takeya H, Nishioka J, Gabazza EC, Suzuki K (1996). β2-glycoprotein I modulates the anticoagulant activity of activated protein C on the phospholipid surface. Thromb Haemost.

[B87] Dunoyer-Geindre S, Kruithof EKO, Galve-deRochemonteix B, Rosnblet C, Gruenberg J, Reber G, deMoerloose P (2001). Localization of β2-glycoprotein 1 in late endosomes of human endothelial cells. Thromb Haemost.

[B88] Esmon CT (2000). The endothelial protein C receptor. Thromb Haemost.

[B89] Dahlback B, Villoutreix BO (2003). Molecular recognition in the protein C anticoagulant pathway. J Thromb Haemost.

[B90] Anderson BD, Bisgaard ML, Lind B, Philips M, Villoutreix BO, Thorsen S (2001). Characterization and structural impact of five novel PROS1 mutations in eleven protein S deficient families. Thromb Haemost.

[B91] Rezende SM, Simmonds RE, Lane DA (2004). Coagulation, inflammation, and apoptosis: different roles for protein S and the protein S-CDb-binding protein complex. Blood.

[B92] Takahashi H, Tatewaki W, Nakamura T, Hanano M, Wada K, Shibata A (1989). Coagulation studies in thrombotic thrombocytopenic purpura, with special reference to von Willebrand factor and protein S. Am J Hematol.

[B93] Oosting J, Derkson RHWM, Bobbink IWG, Hackeng TM, Bouma BN, deGroot PG (1993). Antiphospholipid antibodies directed against a combination of phospholipids with prothrombin, protein C, or protein S: An explanation for their pathogenic mechanisms?. Blood.

[B94] Merrill JT, Zhang HW, Shen C, Butman BT, Jeffries EP, Lahita RG, Myones BL (1999). Enhancement of protein S anticoagulant function by β2-glycoprotein 1, a major target antigen of antiphospholipid antibodies: β2-clycoprotein 1 interferes with binding of protein S to its plasma inhibitor, C4b-binding protein. Thromb Haemost.

[B95] Bick RL, Pegram M (1994). Syndromes of hypercoagulability and thrombosis: a review. Sem Thomb Haemost.

[B96] Duchemin J, Baden-Meunier B, Proulle V, Nouyrigat V, Goujard C, Grimaux M, Devictor D, Tchernia M, Dreyfus M (2001). Lupus anticoagulant occurring during so-called "benign" infectious diseases may be associated with auto-immune transient protein S deficiency and thrombosis. Thromb Haemost.

[B97] Nojima J, Kuratsu H, Suehisa E, Futsukaichi Y, Yamanishi J, Machii T, Iwatani Y, Kanakura Y (2001). Association between the prevalence of antibodies to β2-glycoprotein 1, prothrombin, protein C, protein S, and annexin V in patients with systemic lupus erythematosus and thrombotic and thrombocytopenic complications [with editorial, p985]. Clin Chem.

[B98] Galli M (2000). Should we include anti-prothrombin antibodies in the screening for the antiphospholipid syndrome?. J Autoimm.

[B99] Swadzba J, DeClerck LS, Stevens WJ, Bridts CH, vanCotthem KA, Musial J (1997). Anticardiolipin, anti-beta2-glycoprotein 1, antiprothrombin antibodies, and lupus anticoagulant in patients with systemic lupus erythematosus with a history of thrombosis. J Rheumatol.

[B100] Pengo V, Basiolo A, Brocco T, Torietto S, Ruffati A (1996). Autoantibodies to phospholipid-binding plasma proteins in patients with thrombosis and phospholipid reactive antibodies. Thromb Haemost.

[B101] Madoiwa S, Nakamura Y, Mimuro J, Furusawa S, Koyama T, Sugo T, Matsuda M, Sakata Y (2001). Autoantibody against prothrombin aberrantly alters the proenzyme to facilitate formation of a complex with its physiological inhibitor antithrombin III without thrombin conversion. Blood.

[B102] Pasquier E, Amiral J, Martin LS, Mottier D (2001). A cross-sectional study of anti-phospholipid-protein antibodies in patients with venous thromboembolism. Thromb Haemost.

[B103] Vaarala O, Puurunen M, Manttari M, Manninen V, Aho K, Palosuo T (1996). Antibodies to prothrombin imply a risk of myocardial infarction in middle-aged men. Thromb Haemost.

[B104] Exner T, Kraus M (1999). A monoclonal antibody against prothrombin fragment 1 behaves like a lupus anticoagulant (Letter). Thromb Haemost.

[B105] Galli M, Beretta G, Daldossi M, Bevers RM, Barbui T (1997). Different anticoagulant and immunological properties of anti-prothrombin antibodies in patients with antiphospholipid antibodies. Thromb Haemost.

[B106] Atsumi T, Ieko M, Betolaccini ML, Ichikawa K, Tsutsumi A, Matsuura E, Koike T (2000). Association of autoantibodies against the phosphatidylserine-prothrombin complex with manifestations of antiphospholipid syndrome and with the presence of lupus anticoagulant. Arthritis Rheum.

[B107] Donohoe S, Mackie IJ, Isenberg D, Machin SJ (2001). Anti-prothrombin antibodies: assay conditions and clinical associations in the anti-phospholipid syndrome. Br J Haematol.

[B108] Guerin V, Smith O, White B, Sweetman G, Feighery C, Jackson J (1998). Antibodies to prothrombin in antiphospholipid syndrome and inflammatory disorders. Br J Haematol.

[B109] Raynal P, Pollard HB (1994). Annexins: the problem of assessing the biological role for a gene family of multifunctional calcium- and phospholipid-binding proteins. Biochim Biophys Acta.

[B110] Liemann S, Benz J, Burger A, Voges D, Hofmann A, Huber R, Gottig P (1996). Structural and functional characterization of the voltage sensor in the ion channel human annexin V. Journal of Molecular Biology.

[B111] Nakamura N, Kuragaki C, Shidara Y, Yamaji K, Wada Y (1995). Antibody to annexin V has anti-phospholipid and lupus anticoagulant properties. Am J Hematol.

[B112] Willems GM, Janssen MP, Confurius P, Galli M, Zwaal RF, Bevers EM (2000). Competition of annexin V and anticardiolipin antibodies for binding to phosphatidyl serine containing membranes. Biochemistry.

[B113] Simak J, Holada K, Vostal JG (2002). Release of annexin V-binding membrane microparticles from cultured human umbilical vein endothelial cells after treatment with camptothecin [on-line]. BMC Cell Biol.

[B114] Pasquet J-M, Toti F, Nurden AT, Dachery-Prigent J (1996). Procoagulant activity and active calpain in platelet-derived microparticles. Thromb Res.

[B115] Kuypers FA, Lewis RA, Hua M, Schott MA, Discher D, Ernst JD, Lubin BH (1996). Detection of altered membrane phospholipid asymmetry in subpopulations of human red blood cells using fluorescently labeled annexin V. Blood.

[B116] Dachary-Prigent JD, Freyssinet J-M, Pasquet J-M, Carron J-C, Nurden AT (1993). Annexin V as a probe of aminophospholipid exposure and platelet membrane vesiculation: A flow cytometric study showing a role for free sulfhydryl groups. Blood.

[B117] Boersma AW, Nooter K, Oostrum RG, Stoter G (1996). Quantification of apoptotic cells with fluorescein isothiocyanate-labeled annexin V in Chinese hamster ovary cell cultures treated with cisplatin. Cytometry.

[B118] Savill J, Gregory C, Haslett C (2003). Eat me or die. Science.

[B119] Rauch J, Janoff AS (1996). Antibodies against phospholipids other than cardiolipin: potential roles for both phospholipid and protein. Lupus.

[B120] Donohoe S, Kingdom JCP, Mackie IJ, Burrell S, Quenby S, Jauniaux E, Machin SJ (2000). Ontogony of β2 glycoprotein 1 and annexin V in villous placenta of normal and antiphospholipid syndrome pregnancies. Thromb Haemost.

[B121] Rand JH, Wu XX, Andree HAM, Lockwood CJ, Guller S, Scher J, Harpel PC (1997). Pregnancy loss in the antiphospholipid antibody syndrome – a possible thrombogenic mechanism. N Engl J Med.

[B122] Galli M, Borrelli G, Jacobson EM, Marfisi RM, Finazzi G, Marchioli R, Wisloff F, Marziali S, Morboeuf O, Barbui T (2007). Clinical significance of different antiphospholipid antibodies in the WAPS (warfarin in the antiphospholipid syndrome) study. Blood.

[B123] Matsuda J, Saitoh N, Gohchi K, Gotoh M, Tsukamoto M (1994). Anti-annexin V antibody in systemic lupus erythematosus patients with lupus anticoagulant and/or anticardiolipin antibody [see comments]. Am J Hematol.

[B124] Kuragaki C, Kidoguchi K, Nakamura N, Wada Y (1995). Anti-annexin V antibodies in plasma and serum samples from patients with lupus anticoagulant [Letter on Matsuda et al]. American Journal of Hematology.

[B125] Kaburaki J, Kuwana M, Yamamoto M, Kawai S, Ikeda Y (1997). Clinical significance of anti-annexin V antibodies in patients with systemic lupus erythematosus. Am J Hematol.

[B126] Dubois T, Bisagni-Faure A, Coste J, Mavoungou E, Menkes CJ, Russo-Marie F, Rothhut B (1995). High levels of antibodies to annexins V and VI in patients with rheumatoid arthritis. J Rheumatol.

[B127] Rodriguez-Garcia MI, Fernandez JA, Rodriguez A, Fernandez MP, Gutierrez C, Torre-Alonso JC (1996). Annexin V autoantibodies in rheumatoid arthritis. Ann Rheum Dis.

[B128] Bohm BB, Wilbrink B, Kuettner KE, Mollenhauer J (1994). Structural and functional comparison of anchorin CII (cartilage annexin V) and muscle annexin V. Archives of Biochemistry & Biophysics.

[B129] Mukhopadhyay S, Cho W (1996). Interactions of annexin V with phospholipid monolayers. Biochimica et Biophysica Acta.

[B130] Zhang J, McCrae R (2005). Annexin A2 mediates endothelial cell activation by antiphospholipid/anti-beta-2-glycoprotein I antibodies [with editorial p1845]. Blood.

[B131] Kertesz Z, Yu BB, Steinkasserer A, Haupt H, Benham A, Sim RB (1995). Characterization of binding of human β2-glycoprotein 1 to cardiolipin. Biochem J.

[B132] Arnout J, Wittevrongel C, Vermylen J (1997). Phospholipid binding proteins and the antiphospholipid syndrome: A role for complement factor H?. Thromb Haemost.

[B133] Rampazzo P, Biasiolo A, Garin J, Rosato A, Betterle C, Ruffatti A, Pengo V (2001). Some patients with antiphospholipid syndrome express hitherto undescribed antibodies to cardiolipin-binding proteins. Thromb Haemost.

[B134] Jozsi M, Strobel S, Dahse HM, Liu WS, Hoyer PF, Oppermann M, Skerka C, Zipfel PF (2007). Anti-factor H autoanibodies block C-terminal recognition function of factor H in hemolytic uremic syndrome. Blood.

[B135] Bidot CJ, Jy W, Horstman LL, Huang H, Jimenez JJ, Yaniz M, Ahn YS (2003). Factor VII/VIIa: A new antigen in the antiphospholipid antibody syndrome. Br J Haematol.

[B136] Bidot CJ, Horstman LL, Jy W, Jimenez JJ, Bidot C, Ahn YS, Alexander JS, Gonzalez-Toledo E, Kelley RE, Minagar A (2007). Clinical and neuroimaging correlates of antiphospholipid antibodies in multiple sclerosis. BMC Neurol.

[B137] Kolev K, Lerant I, Skopal J, Keleman A, Nagy Z, Machovich R (2005). Impaired inactivation by antithrombin and hirudin and preserved fibrinogen-clotting activity of thrombin in complex with antithrombin antibody from a patient with antiphospholipid syndrome. Thromb Haemost.

[B138] Shinobu S, Harpel PC, Gharavi A, Rand J, Fillit H (1994). Autoantibodies to heparin from patients with antiphospholipid antibody syndrome inhibit formation of antithrombin III-thrombin formation. Blood.

[B139] Arnout J (1996). The pathogenesis of the anti-phospholipid syndrome: A hypothesis based on parallelisms with heparin-induced thrombocytopenia. Thromb Haemost.

[B140] Cesarman-Maus G, Rios-Luna NP, Deora AB, Huang B, Villa R, Craviota MC, Alarcon-Segovia D, Sanchez-Guerrero J, Hajjar KA (2006). Autoantibodies against the fibrinolytic receptor, annexin 2, in antiphospholipid syndrome [with editorial by K.R. McCrae p4375-6]. Blood.

[B141] Miesbach W, Matthias T, Scharrer I (2005). Identification of thrombin antibodies in patients with antiphospholipid syndrome. Ann N Y Acad Sci.

[B142] Matsuda J, Matsuyama A, Atsumi G, Ohkura N (2008). Sole existence of antithrombin antibody in patients with systemic lupus erythematosus showing tendency of its antigenic determinants directing against exosite II (antithrombin/heparin binding site) of thrombin. Blood Coagul Fibrinolysis.

[B143] Sugi T, McIntyre JA (1995). Autoantibodies to phosphatidylethanolamine (PE) recognize a kininogen-PE complex. Blood.

[B144] Sanmarco M, Alessi MC, Harle JR, Sapin C, Aillaud MF, Gentile S, Juhan-Vague I, Weiller PJ (2001). Antibodies to phosphatidylethanolamine as the only antiphospholipid antibodies found in patients with unexplained thrombosis. Thromb Haemost.

[B145] Bertolaccini ML, Roch B, Armengual O, Atsumi T, Kamashta MA, Hughes GR (1998). Multiple antiphospholipid tests do not increase the diagnostic yield in antiphospholipid syndrome. Br J Rheumatol.

[B146] Drouvalakis KA, Buchanan RR (1998). Phospholipid specificity of autoimmune and drug induced lupus anticoagulants: association of phosphatidylethanolamine reactivated thrombosis in autoimmune disease. J Rheumatol.

[B147] Wu R, Nityanand S, Berglund L, Lithell H, Holm G, Lefvert AK (1997). Antibodies against cardiolipin and oxidatively modified LDL in 50-year-old men predict myocardial infarction. Arterioscler Thromb Vasc Biol.

[B148] Steinerova A, Stozicky F, Racek J, Tatzber F, Zima T, Stetina R (2001). Autoantibodies against oxidized LDL in infants. Clin Chem.

[B149] Yang CD, Hwang KK, Yan W, Gallagher K, FitzGerald J, Grossman JM, Hahn BH, Chen PP (2004). Identification of anti-plasmin antibodies in the antiphospholipid syndrome that inhibit degradation of fibrin. J Immunol.

[B150] Cugno M, Cabibbe M, Galli M, Meroni PL, Caccia S, Russo R, Bottasso B, Mannucci PM (2004). Antibodies to tissue-type plasminogen activator (tPA) in patients with antiphospholipid syndrome: evidence of interaction between the antibodies and the catalytic domain of tPA in 2 patients. Blood.

[B151] Forastiero RR, Maetinuzzo ME, Broze GJ (2003). High titers of autoantibodies to tissue factor pathway inhibitor are associated with the antiphospholipid syndrome. J Thromb Haemost.

[B152] Salemink I, Blezer R, Willems GM, Galli M, Bevers E, Lindhout T (2000). Antibodies to β2-glycoprotein I associated with antiphospholipid syndrome suppress the inhibitory activity of tissue factor pathway inhibitor. Thromb Haemost.

[B153] Barquinero J, Ordi-Ros J, Selva A, Perez-Peman P, Vilardell M, Kamashta M (1993). Antibodies against platelet activating factor in patients with antiphospholipid antibodies. Lupus.

[B154] Vlachoyiannopoulos PG, Mavragani CP, Bourazopoulou E, Balitsari AV, Routsias JG (2004). Anti-CD40 antibodies in antiphospholipid syndrome and systemic lupus erythematosus. Thromb Haemost.

[B155] DeGroot PG, Oosting JD, Derksen RHWM (1993). Antiphospholipid antibodies: specificity and pathophysiology. Bailliere's Clin Hematol.

[B156] Schorer AE, Duane PG, Woods VL, Niewoehner DE (1992). Some antiphospholipid antibodies inhibit phospholipase A2 activity [with editorial pg 10-13]. J Lab Clin Med.

[B157] Rock G, Chauhan K, Jamieson GA, Tandon NN (1994). Anti-CD36 antibodies in patients with lupus anticoagulant and thrombotic complications. Br J Haematol.

[B158] Pelegri Y, Cerrato G, Martinuzzo ME, Careras LO, Forastiero RR (2003). Link between anti-CD36 antibodies and thrombosis in the antiphospholipid syndrome. Clin Exp Rheumatol.

[B159] Rieger M, Mannucci PM, Hovinga JAK, Herzog A, Gerstenbauer G, Konetschny C, Zimmerman K, Scharrer I, Peyvandi F, Galbusera M, Remuzzi G, Bohm M, Pleimauer B, Lammle B, Scheiflinger F (2005). ADAMTS13 autoantibodies in patients with thrombotic microangiopathies and other immunomediated diseases. Blood.

[B160] Gallimore MJ, Jones DW, Winter M (1998). Factor XII determinations in the presence and absence of phospholipid antibodies. Thromb Haemost.

[B161] Jones DW, Mackie IJ, Gallimore MJ, Winter M (2001). Antibodies to factor XII and recurrent fetal loss in patients with the anti-phospholipid syndrome. Br J Haematol.

[B162] Nuss R, Jacobson L, Hathaway WE, Manco-Johnson M, Study Group (1999). Evidence for antiphospholipid antibodies in hemophilic children with factor VIII inhibitors (letter). Thromb Haemost.

[B163] Khamashta MA, Harris EN, Gharavi AE, Derue G, Gil A, Vazquez JJ, Hughes GRV (1988). Immune mediated mechanism for thrombosis antiphospholipid antibody binding to platelet membranes. Ann Rheum Dis.

[B164] Out HJ, deGroot PG, vanVliet M, deGast GC, Nieuwenhuis HK, Derksen RH (1991). Antibodies to platelets in patients with anti-phospholipid antibodies. Blood.

[B165] Martinuzzo ME, Maclouf J, Carreras LO, Levy-Toledano S (1993). Antiphospholipid antibodies enhance thrombin induced platelet activation and thromboxane formation. Thromb Haemost.

[B166] Nozima J, Suehisa E, Kuratsune H, Machi T, Koike T, Kitani T, Kanakura Y, Amino N (1999). Platelet activation induced by combined effects of anticardiolipin and lupus anticoagulant IgG antibodies in patients with systemic lupus erythematosus: Possible association with thrombotic and thrombocytopenic complications. Thromb Haemost.

[B167] Tokita S, Arai M, Yamamoto N, Katagiri Y, Tanoue K, Igarashi K, Umeda M, Inoue K (1996). Specific cross reaction of IgG anti-phospholipid antibody with platelet glycoprotein IIIa. Thromb Haemost.

[B168] Meroni PL, Raschi E, Testoni C, Tincani A, Balestrieri G (2001). Antiphospholipid antibodies and the endothelium. Rheum Dis Clin North Am.

[B169] Pierangeli SS, Espinola RG, Ferrara DE, Harris EN (2001). Activation of endothelial cell nuclear factor kB by antiphospholipid antibodies. Blood.

[B170] Simantov R, LaSala J, Lo S, Gharavi A, Sammaritano L, Salmon J (1995). Activation of cultured vascular endothelial cells by antiphospholipid antibodies. J Clin Invest.

[B171] Simantov R, Lo SK, Gharavi A, Sammaritano LR, Salmon JE, Silverstein RL (1996). Antiphospholipid antibodies activate vascular endothelial cells. Lupus.

[B172] McCrae KR, DeMichele A, Samuels P, Roth D, Kuo A, Meng Q, Rauch J, Cines DB (1991). Detection of endothelial cell-reactive immunoglobulin in patients with antiphospholipid antibodies. Br J Haematol.

[B173] Oosting J, Derksen RH, Blokzijl L, Sixma L, deGroot PG (1992). Antiphospholipid antibody positive sera enhance endothelial cell procoagulant activity – studies in a thrombosis model. Thromb Haemost.

[B174] Carvalho D, Savage CO, Isenberg D, Pearson JD (1999). IgG anti-endothelial cell autoantibodies from patients with systemic lupus erythematosus or systemic vasculitis stimulate the release of two endothelial cell-derived mediators, which enhance adhesion molecule expression and leukocyte adhesion in an autocrine manner. Arthritis Rheum.

[B175] Papa ND, Rascho E, Moroni G, Panzeri P, Borghi MO, Ponticelli C, Tincani A, Balestrieri G, Meroni PL (1999). Anti-endothelial cell IgG fractions from systemic lupus erythematosus patients bind to human endothelial cells and induce a proadhesive and proinflammatory phenotype *in vivo*. Lupus.

[B176] Williams FMK, Parmar K, Hughes GRV, Hunt BJ (2000). Systemic endothelial cell markers in primary antiphospholipid syndrome. Thromb Haemost.

[B177] Porta C, Buggia I, Bonomi I, Caporali R, Scatola C, Montecucco C (1997). Nitrite and nitrate plasma levels, as markers of nitric oxide synthesis, in antiphospholipid antibodies-related conditions and in thrombotic thrombocytopenic purpura (letter). Thromb Haemost.

[B178] Martinuzzo ME, Forastiero RR, Carreras LO (1996). Increased plasma thrombomodulin in different subgroups of patients with antiphospholipid and anti β2 glycoprotein 1 antibodies. Thromb Haemost.

[B179] DelPapa N, Meroni PL, Tincani A, Harris EN, Pierangeli SS, Barcellini W, Borghi MO, Balestreira G, Zanussi C (1992). Relationship between anti-phospholipid and anti-endothelial cell antibodies: further characterization of the reactivity on resting and cytokine-activated endothelial cells. Clin Exp Rheumatol.

[B180] Espinola RG, Liu X, Colden-Stanfield M, Hall J, Harris EN, Pierangeli SS (2003). E-selectin mediates pathogenic effects of antiphospholipid antibodies. J Thromb Haemost.

[B181] Branch DW, Rodgers GM (1993). Induction of endothelial tissue factor activity by sera from patients with antiphospholipid syndrome: A possible mechanism of thrombosis. Am J Obstet Gynecol.

[B182] Atsumi T, Khamashta MA, Amengual O, Hughes GRV (1997). Up-regulated tissue factor expression in antiphospholipid syndrome. Thromb Haemost.

[B183] Jy W, Tiede M, Bidot CJ, Horstman LL, Jimenez JJ, Chirinos J, Ahn YS (2007). Platelet activation rather than endothelial injury identifies risk of thrombosis in subjects positive for antiphospholipid antibodies. Thromb Res.

[B184] Dueymes M, Levy Y, Ziporen L, Jamin C, Piette JC, Shoenfeld Y, Youinou P (1996). Do some antiphospholipid antibodies target endothelial cells?. Ann Med Interne.

[B185] Belizna C, Tervaert JW (1997). Specificity, pathogenicity, and clinical value of antiendothelial cell antibodies. Semin Arthritis Rheum.

[B186] Bordron A, Dueymes M, Levy Y, Jamin C, Leroy JP, Piette JC, Shoenfeld Y, Youinou PY (1998). The binding of some human anti-endothelial cell antibodies induces endothelial cell apoptosis. J Clin Invest.

[B187] Edelsten C, D'Cruz D, Hughes GR, Graham EM (1992). Anti-endothelial cel antibodies in retinal vasculitis. Curr Eye Res.

[B188] Ronda N, Leornado S, Orlandini G, Gatti R, Bellosta S, Bernini F, Borghetti A (1999). Natural anti-endothelial cell antibodies (AECA). J Autoimm.

[B189] Hill MB, Phipps JL, Hughes P, Greaves M (1998). Anti-endothelial cell antibodies in primary antiphospholipid syndrome and SLE: Patterns of reactivity with membrane antigens on microvascular and umbilical venous cell membranes. Br J Haematol.

[B190] Navarro M, Cervera R, Teixido M, Reverter JC, Font J, Lopez-Soto A, Monteagudo J, Escolar G, Ingelmo M (1996). Antibodies to endothelial cells and to β2-glycoprotein I in the anti phospholipid syndrome: prevalence and isotype distribution. Br J Rheumatol.

[B191] deRochemonteix BG, Kobayashi T, Rosnoblet T, Lindsay M, Parton RG, Reber G, deMaistre E, Wahl D, Kruithof EKO, Gruenberg J, deMoerloose P (2000). Interaction of anti-phospholipid antibodies with late endosomes of human endothelial cells. Arterioscler Thromb Vasc Biol.

[B192] Matsuda J, Gotoh M, Gohchi K, Kawasugi K, Tsukamoto M, Saitoh N (1997). Anti-endothelial cell antibodies to the endothelial hybridoma cell line (Eahy926) in systemic lupus erythematosus patients with antiphospholipid antibodies. Br J Haematol.

[B193] Nakamura N, Shidara Y, Kawaguchi N, Azuma C, Mitsuda N, Onishi S, Yamaji K, Wada Y (1994). Lupus anticoagulant autoantibody induces apoptosis in umbilical vein endothelial cells: involvement of annexin V. Biochem Biophys Res Com.

[B194] Shan H, Goldman J, Cunto G, Manuppello J, Chaiken I, Cines DB, Silberstein LE (1998). Heterogeneity of anti-phospholipid and anti-endothelial cell antibodies. J Autoimm.

[B195] Shoenfeld Y (2003). Etiology and pathogenetic mechanisms of the anti-phospholipid syndrome unraveled. Trends Immunol.

[B196] Fischetti F, Durigutto P, Pellis V, Debeus A, Macor P, Bulla R, Bossi F, Ziller F, Sblattero D, Meroni P, Tedesco F (2005). Thrombus formation induced by antibodies to beta-2-glycoprotein-1 is complement dependent and requires a priming factor. Blood.

[B197] Munakata Y, Saito T, Mutsada K, Seino J, Shibata S, Sasoki T (2000). Detection of complement-fixing antiphospholipid antibodies in associated with thrombosis. Thromb Haemost.

[B198] Davis WD, Brey RL (1992). Antiphospholipid antibodies and complement activation in patients with cerebral ischemia. Clin Exp Rheumatol.

[B199] Brekke OH, Michaelsen TE, Sandlie I (1995). The structural requirements for complement activation by IgG: does it hinge on the hinge?. Immunol Today.

[B200] Hinton RC (1994). Neurological symptoms associated with antiphospholipid antibodies. Sem Thomb Haemost.

[B201] Oliver-Minarro D, Sanchez-Ramon S, Rodriguez-Mahou M, Alvarez S, Fernandez-Cruz E (2007). Isolated type 5 antimitochondrial autoantibodies are associated with a history of thrombocytopenia and fetal loss. Fertil Steril.

[B202] Pinckard RN, Olson MS, Kelley RE, DeHeer DH, Palmer JD, O'Rourke RA, Goldfein S (1973). Antibody independent activation of human C1 after interaction with heart subcellular membranes. J Immunol.

[B203] Pinckard RN, Olson MS, Giclas PC, Terry R, Boyer JT, O'Rourke RA (1975). Consumption of classical complement components by heart subcellular membranes in vitro and in patients after acute myocardial infarction. J Clin Invest.

[B204] Kagiyama A, Savage HE, Michael LH, Hanson G, Entman ML, Rossen RD (1989). Molecular basis of complement activation in ischemic myocardium: Identification of the specific molecules of mitochondrial origin that bind human C1q and fix complement. Circ Res.

[B205] Baker WF, Bick RL (1994). Antiphospholipid antibodies in coronary artery disease. Sem Thomb Haemost.

[B206] Baleva M, Boyanovsky B, Nokolov K, Kolarov Z, Nikolova M (1999). High levels of IgA anticardiolipin antibodies in patients with systemic lups erythematosus, Henoch-Schonlein purpura, Sneddon's syndrome and recurrent pregnancy loss (Letter). Thromb Haemost.

[B207] Borzini P, Riva M, Nembri P, Rossi E, Pagliaro P, Vergani P, Greppi P, Tantardini P (1997). CD36 autoantibodies and thrombotic diathesis, thrombocytopenia and repeated early fetal losses. Vox Sang.

[B208] La Rosa L, Meroni PL, Tincani A, Balestrieri G, Faden D, Lojacono A, Morassi L, Brocchi E, Del Papa N, Gharavi A (1994). Beta 2 glycoprotein I and placental anticoagulant protein I in placenta from patients with antiphospholipid syndrome. Journal of Rheumatology.

[B209] Mercier E, Quere I, Mares P, Gris JC (1998). Primary recurrent miscarriages and anti-beta-2-glycoprotein I IgG antibodies induce an acquired activated protein C resistance that can be detected by the modified activated protein C resistance test (Letter). Blood.

[B210] Vora S, Shetty S, Ghosh K (2008). Thrombophilic dimensions of recurrent fetal loss in Indian patients. Blood Coagul Fibrinolysis.

[B211] Ornoy A, Yacobi S, Matalon ST, Blank M, Blumenfeld Z, Miller RK, Shoenfeld Y (2003). The effects of antiphospholipid antibodies obtained from women with SLE/APS and associated pregnancy loss on rat embryos and placental explants in culture. Lupus.

[B212] Salmon JE (2007). Antiphospholipid antibodies revisited: A disorder initiated by inflammation [Theodore E Woodward Award]. Trans Am Clin Climatol Assoc.

[B213] Zwaal RFA, Comfurius P, Bevers EM (1992). Platelet procoagulant activity and microvesicle formation: Its putative role in hemostasis and thrombosis (Review). Biochim Biophys Acta.

[B214] Nomura S, Yanabu M, Fukuroi T, Kido H, Kawakatsu T, Yamaguchi K, Suzuki M, Kokawa T, Yasunaga K (1992). Anti-phospholipid antibodies bind to platelet microparticles in idiopathic (autoimmune) thrombocytopenic purpura. Ann Hematol.

[B215] Combes V, Simon AC, Grau GE, Arnoux D, Camoin L, Sabatier F, Mutin M, Sanmarco M, Sampol J, Dignat-George F (1999). In vitro generation of endothelial microparticles and possible prothrombotic activity in patients with lupus anticoagulant. J Clin Invest.

[B216] Dignat-George F, Camoin-Jau L, Sabatier F, Arnoux D, Anfosso F, Bardin N, Veit V, Combes V, Gentile S, Moal V, Sanmarco M, Sampol J (2004). Endothelial microparticles: a potential contribution to the thrombotic complications of the antiphospholipid syndrome. Thromb Haemost.

[B217] Vallar L, Regnault V, Latger-Cannard V, Lecompte T (2001). Beta 2-glycoprotein I binding to platelet microparticle membrane specifically reduces immunoreactivity of glycoproteins IIb/IIIa. Thromb Haemost.

[B218] Horstman LL, Ahn YS (1999). Platelet microparticles: A wide-angle perspective (Review). Crit Rev Oncol/Hematol.

[B219] Horstman LL, Jy W, Jimenez JJ, Ahn YS (2004). Endothelial microparticles as markers of endothelial dysfunction [Review]. Frontiers in Bioscience.

[B220] Hron G, Kollars M, Binder BR, Eichinger S, Kyrle PA (2006). Identification of patients a low risk for recurrent venous thromboembolism by measuring thrombin generation. JAMA.

[B221] Bidot L, Jy W, Bidot C, Fontana V, Horstman LL, Ahn Y-S (2007). Microparticle-mediated thrombin generation assay: enhanced activity in patients with recurrent thrombosis. J Thromb Haemost.

[B222] Cheng HM (1992). Antibodies to anionic phospholipids in normal human, rabbit, and mouse sera. Thromb Res.

[B223] Santiago MB, Bueno C, Vianna V, Cossermelli W, Oliviera RM (1989). Influence of serum inactivation on detection of anticardiolipin antibodies (ACA) by ELISA. Clin Exp Rheumatol.

[B224] Hasselaar P, Triplett DA, LaRue A, Derksen RHWM, Bookzijl L, deGroot PG, Wagenknecht DR, McIntyre JA (1990). Heat treatment of serum and plasma induces false positive results in the antiphospholipid ELISA. J Rheumatol.

[B225] Matsuda J, Tsukamoto M, Saitoh N, Gochi K, Kinoshita K (1992). Absence of anticardiolipin antibody in non-treated and heat-inactivated normal human sera. Thromb Res.

[B226] McIntyre JA, Wagenknecht DR, Triplett DA (1993). Detection of antiphospholipid antibodies in heat inactivated and normal human sera. Thromb Res.

[B227] Cabiedes J, Cabral AR, Alarcon-Segovia D (1998). Hidden anti-phospholipid antibodies in normal human sera circulate as immune complexes whose antigen can be removed by heat, acid, hypermolar buffers or phospholipase treatment. Eur J Immunol.

[B228] Kra-Oz Z, Lorber M, Shoenfeld Y, Scharff Y (1993). Inhibitor(s) of natural anti-cardiolipin antibodies. Clin Exp Immunol.

[B229] Nguyen DH, Tangvoranuntakul P, Varki A (2005). Effects of natural human antibodies against nonhuman sialic acid that metabolically incorporates into activated and malignant immune cells. J Immunol.

[B230] Cabiedes J, Cabral AR, Lopez-Mendoza AT, Cordero-Esperon HA, Huerta MT, Alarcon-Segovia D (2002). Characterization of anti-phosphatidylcholine polyreactive natural autoantibodies from normal human subjects. J Autoimm.

[B231] Sherer Y, Shoenfeld Y (2000). The idiotypic network in antinuclear-antibody-associated diseases. Int Arch Allergy Immunol.

[B232] Adib M, Ragimbeau J, Avrameas S, Ternynck T (1990). IgG autoantibody activity in normal mouse serum is controlled by IgM. J Immunol.

[B233] Hurez V, Kazatchkine MD, Vassilev T, Ramanathan S, Pashov A, Basuyaux B, deKozak Y, Bellon B, Kaveri SV (1997). Pooled normal human polyspecific IgM contains neutralizing anti-idiotypes to IgG autoantibodies of autoimmune patients and protects from experimental autoimmune disease. Blood.

[B234] Ochsenbein AF, Fehr T, Lutz C, Suter M, Brombacher F, Hengartner H, Zinkernagel RM (1999). Control of early viral and bacterial distribution and disease by natural antibodies. Science.

[B235] Stahl D, Lacroix-Desmazes S, Heudes, Mouthon L, Kaveri SV, Kazatchkine MD (2000). Altered control of self-reactive IgG by autologous IgM in patients with warm autoimmune hemolytic anemia. Blood.

[B236] Moreau A, Lacroix-Desmazes S, Stieltjes N, Saenko E, Kaveri SV, DíOiron R, Sultan Y, Scandella D, Kazatchkine MD (2000). Antibodies to the FVIII light chain that neutralizes FVIII procoagulant activity are present in plasma of nonresponder patients with severe hemophilia A and in normal polyclonal human IgG. Blood.

[B237] Pan ZJ, Anderson CJ, Stafford HA (1998). Anti-idiotypic antibodies prevent the serologic detection of antiribosomal P autoantibodies in healthy adults. J Clin Invest.

[B238] Quan CP, Quan CP, Watanabe S, Pamonsinlapatham P, Bouvet JP (2001). Different dysregulations of the natural antibody repertoire in treated and untreated HIV-1 patients. J Autoimm.

[B239] Shoenfeld Y (1994). Idiotypic induction of autoimmunity: a new aspect of the idiotypic network. FASEB J.

[B240] Fischer P, Jendreyko N, Hoffmann M, Lerch H, Uttenreuther-Fischer MM, Chen PP (1999). Platelet-reactive IgG antibodies cloned by phage display and panning with IVIG from three patients with autoimmune thrombocytopenia. Br J Haematol.

[B241] Yang YY, Fischer P, Leu SI, Zhu M, Woods VL, Chen PP (1999). Possible presence of enhancing antibodies in idiopathic thrombocytopenic purpura. Br J Haematol.

[B242] Yasuda S, Bohgaki M, Atsumi T, Koika T (2005). Pathogenesis of antiphospholipid antibodies: importance of fibrinolysis and monocyte activation via the p38 mitogen-activated protein kinase pathway. Immunobiol.

[B243] Pierangeli SS, Chen PP, Reschi E, Scurati S, Grossi C, Borghi MO, Palomo I, Harris EN, Meroni PL (2008). Antiphospholipid antibodies and the antiphospholipid syndrome: pathogenic mechanisms [with other relevant articles in this special issue and prior one]. Sem Thromb Hemost.

[B244] Galli M, Luciani D, Bertolini G, Barbui T (2003). Lupus anticoagulants are stronger risk factors of thrombosis than anticardiolipin antibodies in the antiphospholipid syndrome: a systematic review of the literature. Blood.

[B245] Matsuura E, Igarashi Y, Yasuda T, Koike T, Triplett DA, Koike T (1994). Anticardiolipin antibodies recognize β2-glycoprotein 1 structure altered by interacting with an oxygen modified solid phase surface. J Exp Med.

[B246] Iverson GM, Matsuura E, Victoria EJ, Cockerill KA, Linnik MD (2002). The orientation of β2GP1 on the plate is important for the binding of anti-β2GP1 autoantibodies by ELISA. J Autoimm.

[B247] Nomura S, Yanabu M, Miyake T, Miyazaki Y, Kido H, Kagawa H, Fukuhara S, Komiyama Y, Matsuura E, Koike T (1995). Relationship of microparticles with beta2-glycoprotein I and P-selectin positivity to anticardiolipin antibodies in immune thrombocytopenic purpura. Ann Hematol.

[B248] Hoefner DM, Yeo KTJ (2002). Lot-to-lot inconsistency of anticardiolipin reagents. Clin Chem.

[B249] Merkel PA, Chang Y, Pierangeli SS, Harris EN, Polisson RP (1999). Comparison between the standard anticardiolipin antibody test and a new phospholipid test in patients with connective tissue disease. J Rheumatol.

[B250] Kilpatrick DC (1998). Factors affecting cardiolipin antibody assays: modification with polyethylene glycol compound. Br J Haematol.

[B251] Reber G, Schousboe I, Tincani A, Sanmarco M, Kveder T, deMoerloose P, Boffa MC, Arvieux J (2002). Inter-laboratory variability of anti-β2-glycoprotein I measurement: Collaborative study in the frame of the European Forum on Antiphospholipid Antibodies Standardization Group. Thromb Haemost.

[B252] Ming CH, Fan YS (1988). Enhancement of anti-phospholipid antibody activity by Tween 20. J Immunol Meth.

[B253] Cabral AR, Cabiedes J, Alarcon-Segovia D (1994). Tween 20 detaches cardiolipin from ELISA plates and makes anticardiolipin antibodies undetectable regardless of the presence of β2-glycoprotein 1. J Immunol Meth.

[B254] Matsuda J, Saitoh N, Gohchi K, Gotoh M, Tsukamoto M (1993). Distinguishing β2-glycoprotein 1 dependent (systemic lupus erythematosus type) and independent (syphilis type) anticardiolipin antibody with Tween 20. Br J Haematol.

[B255] Nava A, Banales JL, Reyes PA (1993). Heat inactivation of bovine serum used for blockade in immunoenzymatic assay is associated with spurious fall on the titers of anticardiolipin antibodies in primary antiphospholipid syndrome sera. J Clin Lab Anal.

[B256] Lockshin MD, Qamar Q, Levy RA, Best MP (1988). IgG but not IgM anti-phospholipid antibody binding is temperature dependent. J Clin Immunol.

[B257] Kamikubo Y, Miyamoto S, Iwasa A, Ishii M, Okajima K (2000). Purification and characterization of factor VII inhibitor found in a patient with life-threatening bleeding. Thromb Haemost.

[B258] Wong RCW, Favaloro EJ (2006). The reactivity of paired plasma and serum samples are comparable in the anticardiolipin and anti-b2-glycoprotein-1 ELISAs: a rebuttal [Response to Lewis et al, JTH 4:265]. J Thromb Haemost.

[B259] Ahn ER, Lander G, Jy W, Bidot C, Jimenez JJ, Horstman LL, Ahn YS (2004). Differences of soluble CD40L in sera and plasma: Implications on CD40L assay as a marker of thrombotic risk. Thromb Res.

[B260] Selva-O'Callaghan A, Ordi-Ros J (1998). IgA anticardiolipin antibodies – relation with other antiphospholipid antibodies and clinical significance. Thromb Haemost.

[B261] Gargulio P, Goldberg J, Romani B, Schiaffini R, Ciampalini P, Faulk WP, McIntyre JA (1999). Qualitative and quantitative studies of autoantibodies to phospholipids in diabetes mellitus. Clin Exp Immunol.

[B262] Wahl DG, Guillemin F, deMaistre E, Perret-Guillaume C, Lecompte T, Thibaut G (1998). Meta-analysis of the risk of venous thrombosis in individuals with antiphospholipid antibodies without underlying autoimmune disease or previous thrombosis. Lupus.

[B263] Wahl DG, Guillemin F, deMaistre E, Perret C, Lecompte T, Thibaut G (1997). Risk for venous thrombosis related to antiphospholipid antibodies in systemic lupus erythematosus, a meta-analysis. Lupus.

[B264] Harris EN, Pierangeli S, Birch D (1994). Anticardiolipin wet workshop report. Fifth international symposium on antiphospholipid antibodies. Am J Clin Pathol.

[B265] Tincani A, Allegri F, Sanmarco M, Cinquini M, Taglietti M, Balestrieri G, Koike T, Ichikawa K, Meroni P, Boffa MC (2001). Anticardiolipin antibody assay: a methodological analysis for a better consensus in routine determinations. A Cooperative Project of the European Antiphospholipid Forum. Thromb Haemost.

[B266] Pierangeli SS, Harris EN (2008). A quarter of a century of in anticardiolipin testing and attempted standardization has led us to here, which is?. Semin Thromb Hemost.

[B267] Fontaine M, Hamburger HA, Nichols WL (2001). Persistent problems with standardization of immunoassays for anti-cardiolipin antibodies (Letter). Thromb Haemost.

[B268] Margolius A, Jacksom DP, Ratnoff OD (1961). Circulating anticoagulants: A study of 40 cases and a review of the literature. Medicine.

[B269] Aggeler PM, Lindsay S, Lucia SP (1946). Studies on the coagulation defect in a case of thrombocytopenic purpura complicated by thrombosis. Am J Pathol.

[B270] Conley CL, Rathbun HK, Morse WI, Robinson JE (1948). Circulating anticoagulants as a cause of hemorrhagic diathesis in man. Bull Johns Hopkins Hosp.

[B271] Bowie EJW, Thompson JH, Jacuzzi CA, Owen CA (1963). Thrombosis in systemic lupus erythematosus despite circulating anticoagulants. J Lab Clin Med.

[B272] Gharavi W, Harris EN, Asherson RA, Hughes GRV (1987). Anti-cardiolipin antibodies: Isotope distribution and phospholipid specificity. Ann Rheum Dis.

[B273] Mueh JR, Herbst KD, Rapaport SI (1980). Thrombosis in patients with the lupus anticoagulant. Ann Int Med.

[B274] Derkens RHWM, Hasselaar P, Blokzijl L, Frits HJ, Myling G, DeGroot PG (1988). Coagulation screen is more specific than the anticardiolipin antibody ELISA in defining a thrombotic subset of patients. Ann Rheum Dis.

[B275] Ferro D, Quintarelli C, Valasini G, Violi F (1993). Lupus anticoagulant and increased thrombin generation in patients with systemic lupus erythematosus. Blood.

[B276] Ghiradello A, Doria A, Ruffatti A, Rigoli AM, Vesco P, Calligaro A, Bambari PF (1994). Antiphospholipid antibodies (aPL) in systemic lupus erythematosus. Are they specific tools for the diagnosis of aPL syndrome?. Ann Rheum Dis.

[B277] Ames PR, Pyke S, Iannaccone L, Brancaccio V (1995). Antiphospholipid antibodies, hemostatic variables and thrombosis – a survey of 144 patients. Thromb Haemost.

[B278] Horbach DA, vanOort E, Donders RCJM, Derksen RHWM, deGroot PG (1996). Lupus anticoagulant is the strongest risk factor for both venous and arterial thrombosis in patients with systemic lupus erythematosus. Comparison between different assays for the detection of antiphospholipid antibodies. Thromb Haemost.

[B279] Roubey RAS, Pratt CW, Buyon JP, Winfield JB (1992). Lupus anticoagulant activity of autoimmune antiphospholipid antibodies is dependent upon β2-glycoprotein 1. J Clin Invest.

[B280] Arvieux J, Pouzol P, Roussel B, Jacob MC, Colomb MG (1992). Lupus-like anticoagulant properties of murine antibodies to beta2-glycoprotein I. Br J Haematol.

[B281] Arnout J, Wittevrongel C, Vermylen J (1997). Murine monoclonal antibodies against beta-2-glycoprotein 1 have lupus anticoagulant activity. Thromb Haemost.

[B282] Willems GM, Janssen MP, Comfurius P, Galli M, Zwaal RF, Bevers EM (2002). Kinetics of prothrombin-mediated binding of lupus anticoagulant antibodies to phosphatidyl serine-containing phospholipid membranes: an ellipsometric study. Biochemistry.

[B283] Takeya H, Mori T, Gabazza EC, Kuroda K, Deguchi H, Matsuura E, Ichikawa K, Koike T, Suzuki K (1997). Anti-β2-glycoprotein I (β2GPI) monoclonal antibodies with lupus anticoagulant-like activity enhance the β2GPI binding to phospholipids. J Clin Invest.

[B284] Bevers EM, Galli M (1992). Cofactors involved in the antiphospholipid syndrome. Lupus.

[B285] Permpikul P, Rao LV, Rapaport SI (1994). Functional and binding studies of the roles of prothrombin and beta 2-glycoprotein I in the expression of lupus anticoagulant activity. Blood.

[B286] Semmelink MJA, Derksen RHWM, Arnout J, deGroot PG (2003). A simple method to discriminate between beta2-glycoprotein I and prothrombin-dependent lupus anticoagulants. J Thromb Haemost.

[B287] Field SL, Hogg PJ, Daly EB, Dai YP, Murray B, Owens D, Chesterman CN (1999). Lupus anticoagulants form immune complexes with prothrombin and phospholipid that can augment thrombin production in flow. Blood.

[B288] Field SL, Chesterman CN, Hogg PJ (2000). Dependence on prothrombin for inhibition of activated protein C activity by lupus antibodies (Letter). Thromb Haemost.

[B289] Rand JH, Wu XX, Giesen P (1999). A possible solution to the paradox of the "lupus anticoagulant": antiphospholipid antibodies accelerate thrombin generation by inhibiting annexin V (Letter). Thromb Haemost.

[B290] Rand JH, Wu XX, Lapinski R, vanHeerde WL, Reutelingsberger CP, Chen PP, Ortel TL (2004). Detection of antibody-mediated reduction of annexin A5 anticoagulant activity in plasma of patients with the antiphospholipid antibody syndrome [Editorial pg 2619]. Blood.

[B291] deLaat B, Wu XX, vanLummel M, Derksen RHWM, deGroot PG, Rand JH (2007). Correlation between antiphospholipid antibodies that recognize domain I o beta2-glycoprotein I and a reduction in the anticoagulant activity of annexin A5. Blood.

[B292] Tripodi A, Mancuso ME, Chantarangkul V, Clerici M, Bader R, Meroni PL, Santagostino E, Mannucci PM (2005). Lupus anticoagulants and their relationship with the inhibitors against coagulation factor VIII: Considerations on the differentiation between the 2 circulating anticoagulants. Clin Chem.

[B293] Asakura H, Ontachi Y, Mizutani T, Kato M, Saito M, Morishita E, Yamazaki M, Suga Y, Takami A, Miyamoto K, Nakao S (2001). Elevated levels of free tissue factor pathway inhibitor antigen in cases of disseminated intravascular coagulation caused by various underlying diseases. Blood Coag Fibrinolysis.

[B294] Ijdo JW, Conti-Kelly AM, Greco P, Abedi M, Amos M, Provenzale JM, Greco TP (1999). Anti-phospholipid antibodies in patients with multiple sclerosis and MS-like illnesses: MS or APS?. Lupus.

[B295] Cuadrado MJ, Khamashta MA, Ballesteros A, Godfrey T, Simon MJ, Hughes GR (2000). Can neurological manifestations of Hughes (antiphospholipid) syndrome be distinguished from multiple sclerosis? Analysis of 27 patients and review of the literature. Medicine (Baltimore).

[B296] Karussis D, Leker RR, Ashkenazi A, Abramsky O (1998). A subgroup of multiple sclerosis patients with anticardiolipin antibodies and unusual clinical manifestations: do they represent a new nosological entity?. Ann Neurol.

[B297] Hughes GRV (1999). The antiphospholipid syndrome and 'multiple sclerosis' [with editorial, pg 109–115]. Lupus.

[B298] Moore PM, Lahita RG (1997). Neuropsychiatric manifestations of system lupus erythematosus. Proceedings of a conference. Ann NY Acad Sci.

[B299] Brey RL, Escalante A (1998). Neurological manifestations of antiphospholipid antibody syndrome. Lupus.

[B300] Miesbach W (2008). Neurological symotoms as a feature of antiphospholipid syndrome. Semin Thromb Hemost.

[B301] Nencini P, Baruffi MC, Abbate R, Massai G, Amaducci L, Inzitari D (1992). Lupus anticoagulant and anticardiolipin antibodies in young adults with cerebral ischemia. Stroke.

[B302] Han MH, Hwang SU, Roy DB, Lundgren DH, Gerlitz B, Robinson WH, Baranzini SE, Grinnell BW, Raine CS, Sobel RA, Han DK, Steinman L (2008). Proteomic analysis of active multiple sclerosis lesions reveals therapeutic targets. Nature.

[B303] Minagar A, Jy W, Jimenez JJ, Mauro LM, Horstman LL, Ahn YS, Sheremata WA (2001). Elevated plasma endothelial microparticles in multiple sclerosis. Neurology.

[B304] Horstman LL, Minagar A, Jy W, Bidot CJ, Jimenez JJ, Ahn YS (2007). Cell-derived microparticles and exosomes in neuroinflammatory conditions [Review]. Int Rev Neurobiol.

[B305] Shoenfeld Y, Sherer Y, Blank M, Asherson RA, Cervera R, Piette JC, Shoenfeld Y (2002). Systemic involvement in the antiphospholipid syndrome: Lessons from animal models. The Antiphospholipid Syndrome II: Autoimmune Thrombosis.

[B306] Shoenfeld Y, Nahum A, Korczyn AD, Dano M, Rabinowitz R, Pick OBCG, Leider-Trajo L, Kalshnikova L, Blank M, Chapman J (2003). Neuronal-binding antibodies from patients with antiphospholipid syndrome induce cognitive deficits following intrathecal passive transfer. Lupus.

[B307] Ziporen L, Shoenfeld Y, Levy Y, Korczyn AD (1997). Neurological dysfunction and hyperactive behavior associated with antiphospholipid antibodies. A mouse model. J Clin Invest.

[B308] Tsukada N, Tanaka Y, Miyagi K, Yanagisawa N, Okana A (1989). Autoantibodies to each protein fraction extracted from cerebral endothelial cell membrane in the sera of patients with multiple sclerosis. J Neuroimmunol.

[B309] Souberbielle BE, Swingler RJ, Davidson DLW, Cull RE, Atkinson S, Davison I, Anderson J, Bell JE, Russell WC (1992). Western blotting analysis in patients with MS using human brain vessels as antigens. Acta Neurol Scand.

[B310] Tintore M, Fernandez AL, Rovira A, Martinez X, Direskeneli H, Kamashta M, Schwartz S, Codina A, Montalban X (1996). Antibodies against endothelial cells in patients with multiple sclerosis. Acta Neurol Scand.

[B311] Trojano M, Defazio G, Ricchiuti F, DeSalvia R, Livrea P (1996). Serum IgG to brain microvascular endothelial cells in multiple sclerosis. J Neurol Sci.

[B312] Noseworthy JH, Lucchinetti C, Rodriguez M, Weinshenker BG (2000). Multiple sclerosis (Review). New Engl J Med.

[B313] Ahn YS, Horstman LL, Jy W, Jimenez JJ, Bowen B (2002). Vascular dementia in patients with immune thrombocytopenic purpura (ITP). Thromb Res.

[B314] Bidot CJ, Jy W, Horstman LL, Jimenez JJ, Yaniz M, Lander G, Ahn YS (2004). Antiphospholipid antibodies in idiopathic thrombocytopenic purpura tend to emerge in exacerbation and decline in remission. Br J Haematol.

[B315] Bidot CJ, Jy W, Horstman LL, Ahn ER, Yaniz M, Ahn YS (2006). Antiphospholipid antibodies (APLA) in immune thrombocytopenic purpura (ITP) and antiphospholipid syndrome (APS). Am J Hematol.

[B316] Schultz DA, Arnold PI, Jy W, Valant P, Gruber J, Ahn YS, Mao FW, Horstman LL (1998). Anti-CD36 autoantibodies in thrombotic thrombocytopenic purpura and other thrombotic disorders: identification of an 85 kd form of CD36 as a target antigen. Br J Hematol.

[B317] Bidot CJ, Jy W, Ortega C, Horstman LL, Bidot C, Petit C, Fontana V, Yaniz M, Ahn Y (2007). Patients with recurrent vs. isolated thrombosis have increased prevalence of anti-CD36 (Gp IV) and elevated endothelial microparticles. Blood.

[B318] Prieto J, Yuste JR, Beloqui O, Cuiveira MP, Riezu JI, Aguirre B, Sangro B (1996). Anticardiolipin antibodies in chronic hepatitis C: Implication of hepatitis C virus as the cause of the antiphospholipid syndrome. Hepatol.

[B319] Constans J, Guerin V, Couchouron A, Seigneur M, Ryman M, Blann AD, Amiral J, Amara A, Peuchant E, Moreau JF, Pellegrini I, Pellegrini JL, Fleury H, Leng B, Conri C (1998). Autoantibodies directed against phospholipids or human beta 2-glycoprotein I in HIV-seropositive patients: relationship with endothelial activation and anti-malonic dialdehyde antibodies. Eur J Clin Invest.

[B320] Zhang R, Lifson JD, Chougnet C (2006). Failure of HIV-exposed CD4+ T cells to activate dendritic cells is reversed by restoration of CD40/CD154 interactions [Editorial p1743]. Blood.

